# Steric control of copper nuclearity in sulfur ligated oxidase mimics alters catechol and phenoxazinone oxidation

**DOI:** 10.1038/s41598-026-60865-4

**Published:** 2026-07-09

**Authors:** Eman I. Khalaf, Fawzya I. Elshami, Shaimaa F. Gad, Amr M. Beltagi, Ali M. Nassar, Shaban Y. Shaban

**Affiliations:** Chemistry Department, Faculty of Science, Kafrelsheikh University, Kafrelsheikh, 33516 Egypt

**Keywords:** Copper nuclearity control, Sulfur-ligated complexes, Catechol oxidase mimic, Phenoxazinone synthase, Steric tuning, Bioinorganic catalysis, Dicopper cooperativity, Stopped-flow kinetics, Biochemistry, Chemical biology, Chemistry

## Abstract

Control over copper nuclearity is a key issue in bioinspired oxidation chemistry, yet its mechanistic consequences in sulfur-donor environments remain insufficiently understood. Here, remote tert-butyl substitution on a flexible dithioether-dithiolate ligand scaffold switches the preferred copper(II) assembly from binuclear [CuS_4_]_2_ to mononuclear [CutBuS_4_], enabling a direct assessment of nuclearity effects within a common sulfur-ligated framework. Combined spectroscopic, electrochemical, stopped-flow kinetic, and DFT studies show that this steric perturbation modifies complex stability, productive substrate binding, and the extent to which catalytic turnover benefits from metal-metal cooperativity. In the aerobic oxidation of 3,5-di-tert-butylcatechol, both complexes follow a two-step sequence of rapid reversible substrate binding and slower oxidation, and both reach similar maximum turnover frequencies under saturating conditions, ca. 1700–1750 h^− 1^. The binuclear [CuS_4_]_2_ nevertheless shows stronger productive substrate binding, with *K*_*M*_ values of 3.4 vs. 5.0 mM for the mononuclear $\:\left[CutBu{S}_{4}\right]$, respectively. In contrast, oxidation of o-aminophenol to aminophenoxazinone is strongly nuclearity-dependent, with [CuS_4_]_2_ displaying substantially higher activity than $\:\left[CutBu{S}_{4}\right]$ (2772 vs. 684 h^− 1^), consistent with a decisive role for dicopper cooperativity in oxidative coupling. Detection of H_2_O_2_ and the lack of 4-nitrocatechol oxidation support an oxidase-type pathway in which catalytic competence depends on substrate reducing power and access to productive oxygen-dependent redox chemistry.

**Supplementary Information:**

The online version contains supplementary material available at 10.1038/s41598-026-60865-4.

## Introduction

Copper-containing enzymes play central roles in aerobic oxidation chemistry, particularly in processes involving activation of molecular oxygen at multinuclear copper sites. Catechol oxidase (CO) and phenoxazinone synthase (PHS) are especially informative in this context because they catalyse the oxidation of phenolic and aminophenolic substrates through copper-mediated, oxygen-dependent pathways and thus provide valuable reference points for biomimetic oxidation chemistry^1,2^. In CO, a coupled binuclear copper centre promotes the oxidation of *o*-diphenols to the corresponding *o*-quinones through redox cycling involving oxygen-derived dicopper intermediates^3–7^. PHS likewise exploits multiple copper centres to mediate the oxidative coupling of *o*-aminophenols to phenoxazinone products through sequential electron-transfer and bond-forming steps. Although these enzymes operate on different substrate classes, both illustrate how copper nuclearity and cooperativity can shape substrate activation and aerobic oxidation reactivity^2,8–11^. Synthetic models of these systems have provided important insight into the relationship between ligand environment, metal nuclearity, and catalytic oxidation behavior^7,12^. A wide range of N- and O-donor copper complexes has been explored as catechol oxidase and phenoxazinone synthase mimics, including dinuclear systems active in the aerobic oxidation of 3,5-di-tert-butylcatechol (DTBC) to 3,5-di-tert-butyl-1,2-benzoquinone (DTBQ)^13,14^ and mono- as well as polynuclear complexes that promote oxidative coupling of *o*-aminophenol (OAP) to 2-aminophenoxazin-3-one (APX)^15–17^. However, systems that combine controllable nuclearity, dual CO/PHS-type reactivity, and a sufficiently conserved ligand environment to allow meaningful mechanistic comparison remain relatively uncommon.

Sulfur-donor ligands are particularly attractive for addressing this problem because they can stabilize reduced copper states, modulate Cu^II^/Cu^I^ redox behaviour, and influence oxygen-dependent reactivity in ways not easily reproduced by purely nitrogen-donor frameworks^18,19^. Among such systems, the flexible ligand [1,2-bis(2-mercaptophenylthio)ethane]^2−^ (*S*_4_^2−^) and its sterically demanding analogue [1,2-bis(3,5-di-tert-butyl-2-mercaptophenylthio)ethane]^2−^ (*tBuS*_4_^2−^) provide a particularly useful design pair for probing nuclearity effects within a common sulfur-rich donor framework^20,21^. The less hindered ligand favors dinuclear copper assemblies with Cu⋯Cu separations compatible with cooperative reactivity, whereas remote tert-butyl substitution disfavors close metal-metal approach and shifts the system toward mononuclear coordination^12,22–27^. This steric differentiation therefore offers a controlled strategy for evaluating how copper nuclearity influences productive substrate binding, turnover, and oxidation pathway without fundamentally changing donor type^12,22–27^.

**Scheme 1:**
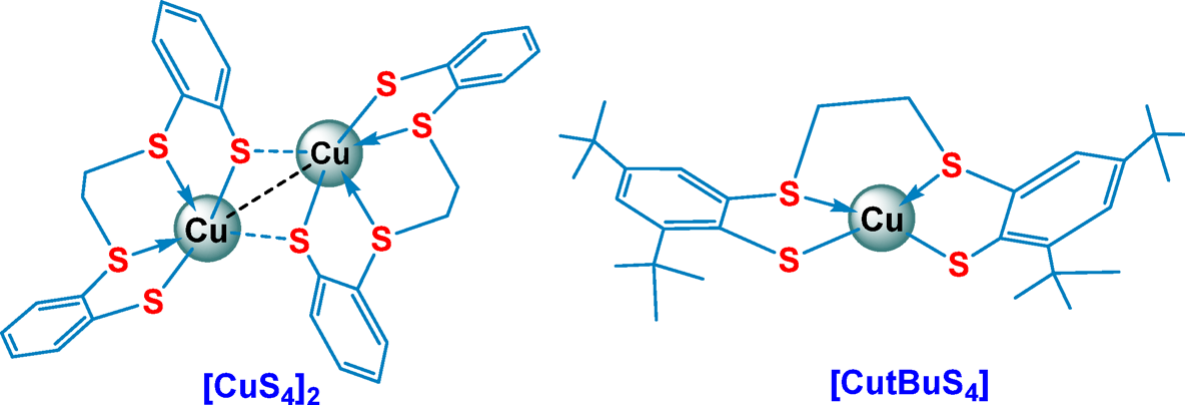
Structures of the binuclear [CuS_4_]_2_ and mononuclear [CutBuS_4_] complexes showing the effect of tert-butyl groups on switching copper nuclearity.

In the present work, and as part of our continuing efforts in biomimetic copper oxidation chemistry^28–32^, we investigate two closely sulfur‑ligated copper(II) complexes derived from these flexible ligands: the binuclear [CuS_4_]_2_ and the mononuclear [CutBuS_4_] (Scheme 1). Building on literature reports that catalytic efficiency in Cu(II) catechol oxidase models often correlates with electrochemical properties, but is strongly modulated by ligand environment, nuclearity, and solvent, these complexes provide a sulfur‑rich platform in which remote steric modification controls nuclearity while preserving the primary donor set^23,24^. Using spectroscopic, electrochemical, stopped‑flow kinetic, and DFT methods, we show that this steric perturbation switches the preferred copper assembly and, in doing so, alters complex stability, productive substrate binding, and the extent to which catalytic turnover benefits from metal–metal cooperativity. Both complexes efficiently catalyse the aerobic oxidation of DTBC, but they diverge more strongly in the oxidation of OAP to APX, where the binuclear system displays a marked kinetic advantage. These findings define a direct relationship between sterically controlled nuclearity and oxidation mechanism in sulfur‑ligated copper systems and show that remote ligand tuning can distinguish between pathways governed primarily by substrate binding and those that require cooperative dicopper reactivity.

## Experimental section

### Materials and methods

All chemicals were of reagent grade and used as received unless otherwise stated. 3,5-DTBC, 1,2-dibromoethane, copper(II) nitrate monohydrate Cu(NO_3_)_2_·H_2_O, 4-nitrocatechol, and OAP were purchased from Sigma-Aldrich. Solvents were dried and distilled under nitrogen before use. Dynamic light scattering (DLS) measurements were performed on a Nanoplus analyzer to assess particle size distributions after 5 min sonication in quartz cuvettes. Infrared spectra were recorded on a Thermo Scientific Nicolet iS50 FTIR spectrometer using KBr pellets (400–4000 cm^−1^). UV–Vis spectra were measured on a PEAK USA model C7000V spectrophotometer in the range 190–1100 nm. Electrospray ionization mass spectra (ESI-MS) were obtained on a Waters Xevo G2-XS QTof mass spectrometer. Surface morphology and elemental composition were examined using a Jeol JSM-T300 scanning electron microscope (SEM) equipped with energy-dispersive X-ray spectroscopy (EDS); samples were drop-cast onto glass slides and sputter-coated with platinum to ensure conductivity.

### Synthesis

#### S_4_H_2_

The ligand S_4_H_2_ was synthesized according to a reported procedure with slight modification^20,21^. To a stirred solution of 2-mercaptothiophenol (5.00 g, 35.6 mmol) in dry dimethylformamide (DMF, 50 mL) under nitrogen was added potassium carbonate (K_2_CO_3_, 9.85 g, 71.2 mmol). The mixture was stirred at room temperature for 30 min, after which 1,2-dibromoethane (3.34 g, 17.8 mmol) dissolved in DMF (20 mL) was added dropwise. The reaction mixture was heated to 60 °C and stirred under nitrogen for 12 h. After cooling to room temperature, the mixture was poured into ice-cold water (200 mL), extracted with dichloromethane (3 × 50 mL), dried over anhydrous Na_2_SO_4_, and concentrated under reduced pressure to afford S_4_H_2_ as a pale yellow solid. Yield: 4.90 g (89%). ^1^H NMR (400 MHz, CDCl_3_, *δ*, ppm): 7.45–7.38 (m, 4 H, Ar-H), 7.25–7.18 (m, 4 H, Ar-H), 3.52 (s, 2 H, SH), 2.98 (s, 4 H, CH_2_). IR (KBr, cm^− 1^): 2550 (w, S-H), 1580 (s, C = C), 750 (s, C-S). ESI-MS (*m*/*z*): [*M*_2_−*H*]^+^ 617.04 (calcd 616.98).

#### tBuS_4_H_2_

The sterically hindered ligand tBuS_4_H_2_ was prepared following a modified procedure based on Sellmann’s protocol^20,21^. 3,5-Di-*tert*-butyl-2-mercaptothiophenol (8.40 g, 35.6 mmol) was dissolved in dry DMF (60 mL) under nitrogen, and K_2_CO_3_ (9.85 g, 71.2 mmol) was added. After stirring at room temperature for 30 min, a solution of 1,2-dibromoethane (3.34 g, 17.8 mmol) in DMF (20 mL) was added dropwise. The mixture was heated to 70 °C and stirred for 15 h. After cooling, the reaction mixture was poured into ice-cold water (250 mL), extracted with dichloromethane (3 × 60 mL), dried over Na_2_SO_4_, and concentrated to dryness to give tBuS_4_H_2_ as a white crystalline solid. Yield: 8.02 g (80%). ^1^H NMR (400 MHz, CDCl_3_, *δ*, ppm): 7.32 (d, 2 H, Ar-H), 7.18 (d, 2 H, Ar-H), 3.65 (s, 2 H, SH), 3.05 (s, 4 H, CH_2_), 1.42 (s, 18 H, C(CH_3_)_3_), 1.28 (s, 18 H, C(CH_3_)_3_). IR (KBr, cm^− 1^): 2560 (w, S-H), 2950 (m, C-H), 1590 (s, C = C), 740 (s, C-S). ESI-MS (*m*/*z*): 1066.59 [*M*_2_−*H*]^+^ (calcd 1065.84).

#### Binuclear [CuS_4_]_2_

To a solution of S_4_H_2_ (0.31 g, 1.0 mmol) in methanol (20 mL) under nitrogen, triethylamine (0.28 mL, 2.0 mmol) was added. After stirring for 15 min, Cu(NO_3_)_2_·H_2_O (0.41 g, 2.0 mmol) in methanol (10 mL) was added dropwise. The mixture was stirred at room temperature for 6 h, forming a dark green precipitate. The solid was filtered, washed with cold methanol and diethyl ether, and dried under vacuum to yield dark green crystals (0.39 g, 84%). IR (KBr, cm^−1^): 1575 (s, C = C), 735 (s, C-S), absence of S-H stretch at ~ 2550 cm^−1^.

#### Mononuclear [CutBuS_4]_^]^

The complex [CutBuS_4_] was synthesized using a similar procedure. tBuS_4_H_2_ (1.14 g, 2.0 mmol) was dissolved in methanol (25 mL) under nitrogen, and triethylamine (0.28 mL, 2.0 mmol) was added to deprotonate the thiol groups. After stirring for 15 min, Cu(NO_3_)_2_·H_2_O (0.41 g, 2.0 mmol) in methanol (10 mL) was added dropwise. The mixture was stirred at room temperature for 8 h, followed by heating at 60 °C for 4 h, forming a dark green precipitate. The solid was filtered, washed with cold methanol (10 mL) and diethyl ether (10 mL), and dried under vacuum as dark green crystals. Yield: 0.65 g (55%). IR (KBr, cm^−1^): 2955 (m, C-H), 1585 (s, C = C), 730 (s, C-S), absence of S-H stretch at ~ 2560 cm^−1^. MS (m/z): 596.57 [M]⁺ (calcd. 596.47).

### Cyclic voltammetry measurements

Cyclic voltammetry (CV) experiments were performed using a PalmSens 4 potentiostat/galvanostat controlled with PS Trace 5.9 software. A three-electrode microcell was employed, consisting of a carbon paste electrode (CPE; BAS MF-2010, 3 mm diameter) as the working electrode, an Ag/AgCl (3 M KCl) electrode as the reference electrode, and a platinum wire as the counter electrode. The carbon paste was prepared by thoroughly mixing graphite powder (5.0 g, particle size 1–2 *µ*m) with Nujol oil (1.8 mL) in an agate mortar until a homogeneous paste was obtained. The resulting paste was packed into the electrode cavity, and the electrode surface was polished with clean paper to obtain a smooth and shiny finish. Before each measurement, the working electrode was conditioned by recording 10 CV scans between + 1.2 and − 0.6 V in acetate buffer (pH 5.0) at a scan rate of 100 mV s^− 1^. All measurements were carried out at room temperature in deaerated acetate buffer solution (pH 5.0).

### Computational methodology

All quantum-chemical calculations were performed using the Gaussian 09 software package. Geometry optimizations of the free ligands S_4_H_2_ and tBuS_4_H_2_, together with their copper(II) complexes [CuS_4_]_2_ and [CutBuS_4_], were carried out using the hybrid exchange-correlation functional PBE1PBE (PBE0)^33–35^ in combination with the def2-SVP basis set^36–38^. This level of theory was selected because it provides a balanced description of metal-sulfur bonding, steric effects, and electronic structure in transition-metal thiolate complexes. All plausible isomeric forms were considered, and geometry optimizations were performed in the gas phase. The nature of each stationary point was verified by analytical frequency calculations at the same level of theory, and all optimized structures were confirmed as true minima by the absence of imaginary frequencies.

The electronic states and spin multiplicities were explicitly considered in all calculations. The free ligands S_4_H_2_ and tBuS_4_H_2_ were optimized as closed-shell singlet species (charge = 0, multiplicity = 1). The neutral mononuclear Cu(II) complexes [CuS_4_] and [CutBuS_4_] were treated as doublet systems (charge = 0, multiplicity = 2), consistent with a single *d*^9^ Cu(II) center. For the dicopper(II) species [CuS_4_]_2_ and the hypothetical [CutBuS_4_]_2_ dimer, both the antiferromagnetically coupled broken-symmetry singlet (charge = 0, multiplicity = 1) and the ferromagnetically coupled triplet state (charge = 0, multiplicity = 3) were optimized independently using unrestricted DFT. The singlet–triplet energy separation Δ*E*_S–T_ = *E*_triplet_−*E*_singlet_ was evaluated from the calculated electronic energies to assess magnetic coupling within the dicopper core.

Frontier molecular orbital (FMO) energies (HOMO and LUMO) were extracted from the optimized geometries to compute the HOMO–LUMO energy gap (ΔE_gap_) and global reactivity descriptors: chemical hardness (η), softness (S), electronegativity (χ), electrophilicity index (ω), and nucleophilicity index (N)^39,40^. Molecular electrostatic potential (MEP) surfaces were generated to visualize electron density distribution and identify electrophilic (negative potential) and nucleophilic (positive potential) regions^41^. Orbital plots and MEP isosurfaces were visualized using Chemcraft^42^ and GaussView^43^. Time-dependent density functional theory (TD-DFT) calculations were performed at the TD-PBE1PBE/def2-SVP level to simulate the electronic absorption spectra in DMF solution (PCM model). The 20 lowest singlet excited states were computed for each system. Excitation energies, oscillator strengths (*f*), and dominant orbital contributions were extracted and processed with GaussSum^44^ to generate simulated UV–Vis spectra (Gaussian broadening, FWHM = 0.3 eV) over 200–800 nm. These theoretical spectra were directly compared with experimental data to assign electronic transitions (ligand-centered, LMCT, and charge-transfer).

### Electron paramagnetic resonance measurements

Continuous‑wave X‑band electron paramagnetic resonance (EPR) spectra were recorded on a Bruker EMX EPR spectrometer (Bruker, Germany) equipped with a standard rectangular cavity (ER 4102) at the National Center for Radiation Research and Technology (NCRRT). The EPR signals of the generated paramagnetic species were measured at room temperature using polycrystalline samples of [CuS_4_]_2_ and [CutBuS_4_] packed in quartz tubes. The g values were obtained from field positions calibrated with a standard Cu(II) marker measured under identical conditions.

### Biomimetic catalytic activities: experimental procedures

*2.6.1. Catechol Oxidase-like Activity.* The catechol oxidase-like activity of [CuS_4_]_2_ and [CutBuS_4_] was evaluated through the aerobic oxidation of 3,5-di-*tert*-butylcatechol (3,5-DTBC) to 3,5-di-*tert*-butyl-1,2-benzoquinone (3,5-DTBQ) in methanol at 25 ± 0.1 °C under ambient atmosphere. Reactions were monitored spectrophotometrically by following the growth of the quinone absorption band at *λ*_max_ = 400 nm (*ε* = 1900 M^−1^cm^− 1^). Typical reaction conditions were 5 × 10^− 5^ M complex and 5 × 10^− 3^ M 3,5-DTBC (100 equiv) in air-saturated methanol (3 mL). Absorbance was recorded every 30 s for 30–120 min using a temperature-controlled UV-Vis spectrophotometer. Blank experiments performed in the absence of catalyst or under anaerobic conditions showed negligible background oxidation.

Detailed kinetics was performed on an Applied Photophysics SX20 stopped-flow spectrometer equipped with a diode-array detector. Pseudo-first-order conditions were used ([3,5-DTBC] = 0.5–6.0 mM, [complex] = 2.5 × 10^−^⁵ M after mixing, methanol, 25 °C). Absorbance-time traces at 400 nm were fitted globally to a two-step sequential model (fast reversible binding → slow catalytic oxidation) using Pro-Data Viewer software. Michaelis-Menten parameters (K_M_, V_max_) were derived from the steady-state phase. Hydrogen peroxide production was quantified using the iodide assay. To 5 × 10^−^⁵ M complex + 5 × 10^−^³ M 3,5-DTBC in methanol were added excess KI (0.2 M) and catalytic (NH_4_)₆Mo₇O_24_ (10^−^⁴ M). The growth of I₃^−^ at 351 nm (ε = 25 700 M^−1^ cm^−1^) was monitored; yield was calculated as equiv I₃^−^ per equiv quinone formed.

#### Phenoxazinone synthase-like activity

The phenoxazinone synthase-like activity was assessed by the aerobic oxidative coupling of o-aminophenol (OAP) to 2-aminophenoxazin-3-one (APX) in methanol at 25 ± 0.1 °C. The reaction was followed by the growth of the APX band at λmax ≈ 430 nm (ε ≈ 16 500 M^−1^ cm^−1^). Typical conditions: 5 × 10^−^⁵ M complex + 5 × 10^−^³ M OAP (100 equiv) in air-saturated methanol (3 mL). Spectra were recorded every 30 s for 60–180 min. Controls (no catalyst or anaerobic) showed no significant product formation. Kinetics were measured on the same stopped-flow instrument under pseudo-first-order conditions ([OAP] = 0.5–6.0 mM, [complex] = 2.5 × 10^−^⁵ M after mixing, methanol, 25 °C). Traces at 430 nm were fitted to a sequential model (fast coordination → slow oxidative coupling). Michaelis-Menten parameters were obtained from the steady-state phase. All experiments were performed in triplicate; errors are reported as standard deviations.

## Results and discussion

### Steric control of copper nuclearity

**Scheme 2:**
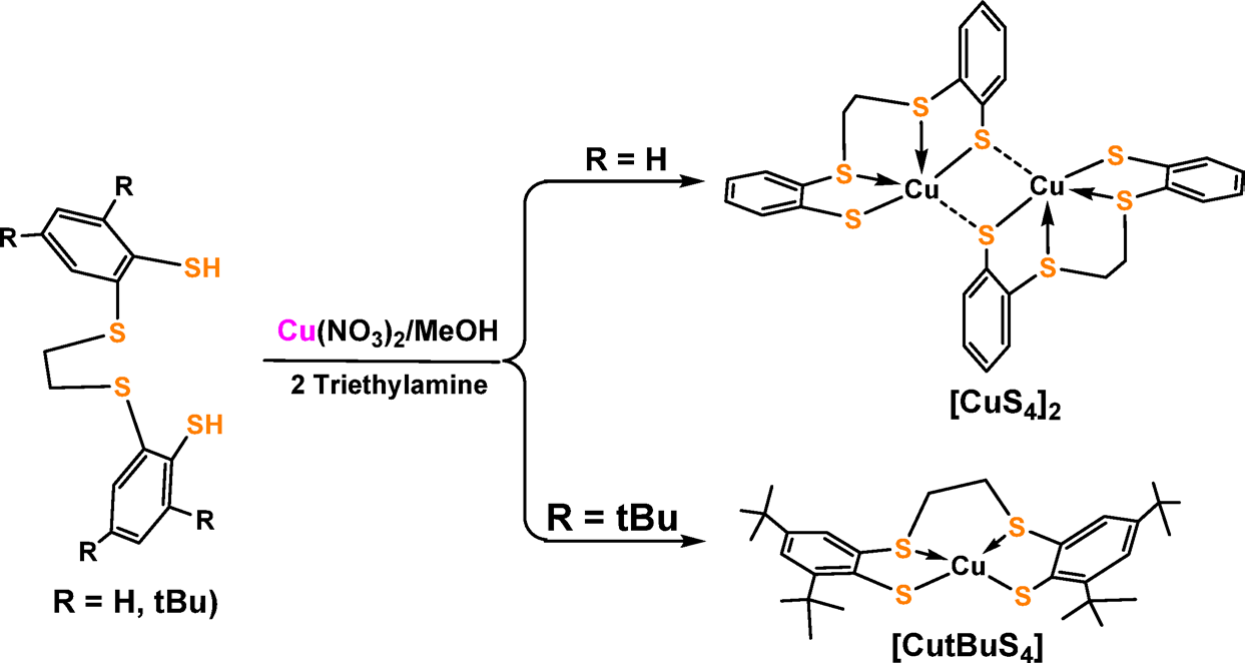
Synthesis of [CuS_4_]_2_ and [CutBuS_4_] complexes.

The ligand precursors $\:{S}_{4}{H}_{2}$ and $\:tBu{S}_{4}{H}_{2}\:$were prepared by modified literature procedures involving nucleophilic substitution of 1,2-dibromoethane with the corresponding mercaptophenyl thiolates under basic DMF conditions^20,21^. This ligand pair preserves a common sulfur-donor framework while introducing a defined steric perturbation through remote tert-butyl substitution. Upon reaction with Cu(NO_3_)_2_, the less hindered S_4_^2−^ ligand preferentially gives binuclear copper species featuring rhomboidal [CuS_4_]_2_ cores sustained by bridging thiolate donors, whereas the sterically encumbered tBuS_4_^2−^-analogue favours formation of the mononuclear complex [CutBuS_4_)] by suppressing close Cu⋯Cu approach and extensive metal bridging^45^. This steric differentiation therefore provides a controlled basis for assessing how copper nuclearity influences the structural, electronic, and catalytic properties of closely related sulfur-ligated complexes. The mononuclear complex [CutBuS_4_] (Hayam-L2) exhibits a dominant ion at m/z 596.57, in excellent agreement with the calculated molecular ion (M⁺, calcd 596.47), confirming the proposed mononuclear formulation (Fig. S1, Supporting Information). For the S_4_-based system, the spectrum displays a strong ion at m/z 617.04, consistent with a dimeric ligand species derived from S_4_H_2_, together with higher-m/z ions assignable to Cu–S_4_ aggregates built from the [CuS_4_] and [CuS_4_]_2_ units. These qualitative mass-match relationships are in line with the proposed dicopper formulation based on the S_4_^2−^ scaffold (Scheme 2; Fig. S2, Supporting Information).

**Fig. 1:**
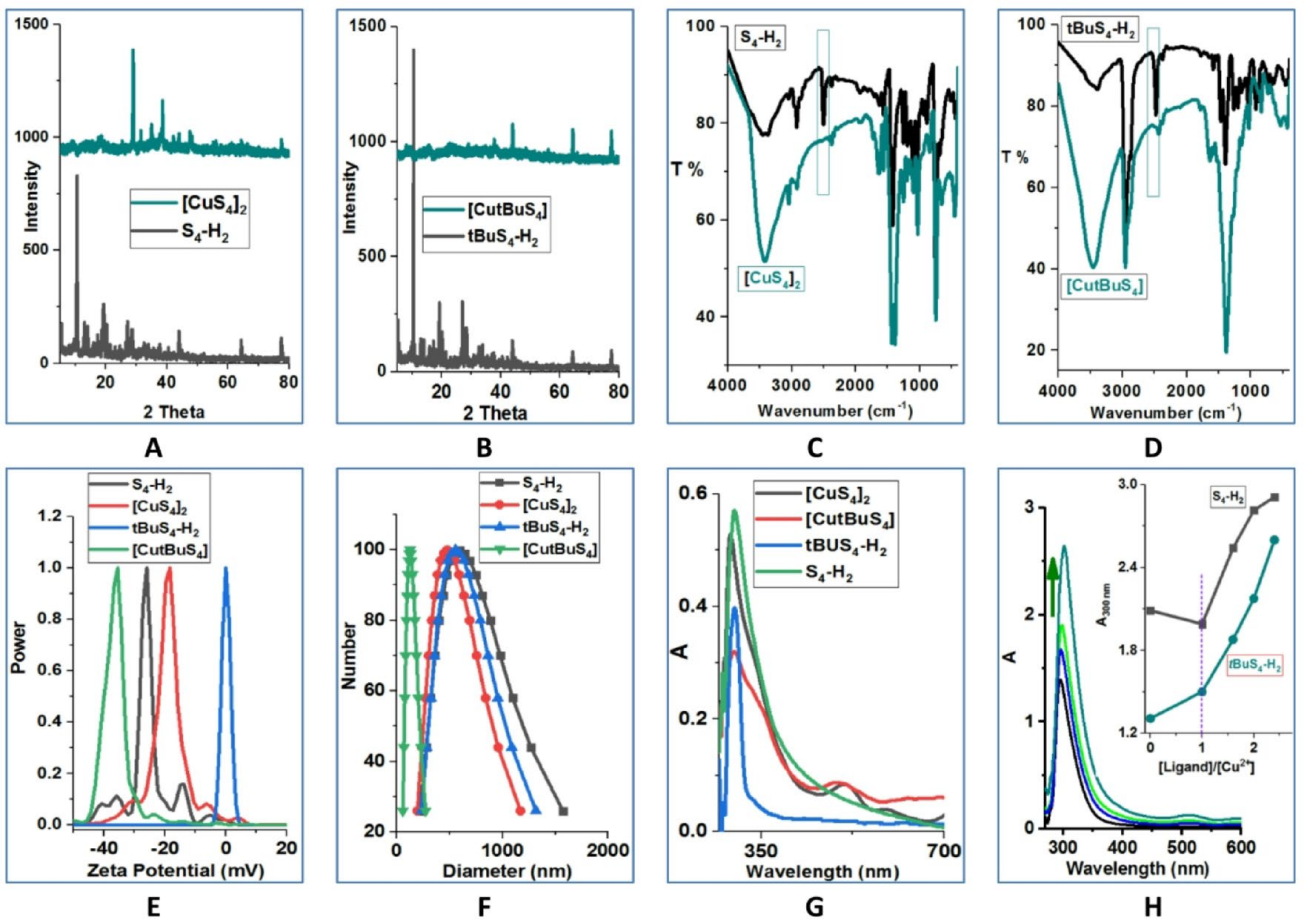
XRD patterns of (**A**) S_4_^2−^ and its copper complex and (**B**) tBuS_4_^2−^ and its copper complex; IR (KBr) spectra of (**C**) S_4_H_2_ and its Cu(II) complex and (**D**) tBuS_4_H_2_ and its Cu(II) complex; (**E**) zeta-potential distributions; (**F**) DLS size distributions; (**G**) UV–Vis spectra of all materials; and (**H**) Job’s titration plot for the [Cu(tBuS_4_)] system at 300 nm.

FT-IR spectroscopy provides evidence for copper-thiolate coordination and for the distinct structural consequences of steric bulk in the two systems (Fig. 1C, D). The free ligand precursors $\:{S}_{4}{H}_{2}$ and $\:tBu{S}_{4}{H}_{2}$ exhibit S-H stretching bands at 2504 and 2481 cm^− 1^, respectively, together with C-S stretching vibrations at 741 and 734 cm^− 1^. Upon complexation, the S-H bands disappear and the C-S vibrations shift to higher frequency, to 754 cm^− 1^ for the S_4_-H_2_ derived complex and 743 cm^− 1^ for the tBuS_4_-H_2_ derived complex, consistent with deprotonation and coordination of the thiolate donors to copper. In the complex derived from the less hindered ligand, broader features and the appearance of additional Cu-S vibrations in the 400–450 cm^− 1^ region are consistent with bridged sulfur coordination in a binuclear environment. By contrast, the tert-butyl-substituted complex exhibits narrower and less congested vibrational features, supporting a more discrete and sterically protected coordination environment consistent with predominantly mononuclear copper binding^46^.

Powder X-ray diffraction further supports the structural consequences of ligand steric bulk (Fig. 1A, B). The free ligand based on the less hindered framework displays broad reflections at approximately 2*θ* = 15°, 25°, and 35°, consistent with polycrystalline aggregation, whereas the tert-butyl-substituted analogue shows weaker and broader features, indicating reduced packing efficiency and greater amorphous character. After copper coordination, both systems exhibit sharper reflections, but the complex derived from the unsubstituted ligand more readily presents patterns consistent with dinuclear or oligomeric copper-thiolate assemblies, whereas the sterically encumbered analogue shows a more regular diffraction profile consistent with a more discrete, nuclearity-limited species. Overall, the PXRD data support the conclusion that remote steric bulk suppresses aggregation and disfavors higher-nuclearity assembly^47^.

Dynamic light scattering and zeta-potential measurements further show that remote steric bulk strongly influences the solution behavior of these sulfur-ligated systems in methanol (Fig. 1E, F; Table 1). The free ligands S_4_-H_2_ and tBuS_4_-H_2_ both form relatively large aggregates, with Z-average diameters of 595 and 556 nm, respectively, but differ markedly in surface charge. Upon copper coordination, the difference becomes more pronounced: [CuS_4_]_2_ retains a comparatively large hydrodynamic diameter of 478 nm, whereas the tert-butyl-substituted complex [Cu(tBuS_4_)] forms much smaller particles with an average diameter of 125 nm and the most negative zeta potential in the series^48^. These data indicate that remote tert-butyl substitution not only limits aggregation but also enhances colloidal stabilization, consistent with the formation of a more discrete copper species in solution.

UV-Vis spectroscopy reveals distinct electronic signatures for the two copper systems that support their different nuclearities (Fig. 1G). The free ligand precursors $\:{S}_{4}{H}_{2}$ and $\:tBu{S}_{4}{H}_{2}$ both exhibit intense *π*→*π*∗ transitions near 300 nm characteristic of aromatic conjugation. Upon copper coordination, the S_4_-H_2_ derived complex displays LMCT and d-d bands at 335, 509, and 589 nm, consistent with dinuclear copper-sulfur assemblies. In contrast, the tBuS_4_-H_2_ derived complex shows red-shifted absorptions at 339, 501, and 631 nm, consistent with sterically protected mononuclear copper centers. These spectral features align well with those reported for related Cu-thiolate proteins and synthetic complexes, which typically show strong ligand-centered UV transitions together with LMCT and d-d bands in the visible region for square-planar or distorted tetrahedral Cu^II^ environments^49,50^.

Stoichiometric characterization by Job’s method confirms a 1:1 metal-to-ligand binding ratio for both ligand systems. UV-Vis titrations at pH 7.0 show progressive increases in absorbance near 300 nm upon incremental addition of Cu^II^, consistent with formation of copper complexes stabilized by the tetradentate thiolate ligands (Fig. 1H). The sterically hindered tBuS_4_^2−^ system exhibits slight bathochromic shifts relative to the S_4_^2−^ complex, reflecting the electronic influence of the tert-butyl substituents, while maintaining the same 1:1 binding stoichiometry^11,51,52^. These spectroscopic results, taken together with the PXRD, FT-IR, and UV-Vis data, demonstrate that remote steric modification from S_4_^2−^ to tBuS_4_^2−^ systematically influences the preferred copper nuclearity and coordination environment while preserving the fundamental metal-to-ligand stoichiometry. The less hindered ligand favors binuclear assemblies with bridging thiolate coordination, whereas the bulkier ligand supports more discrete mononuclear species.

**Fig. 2:**
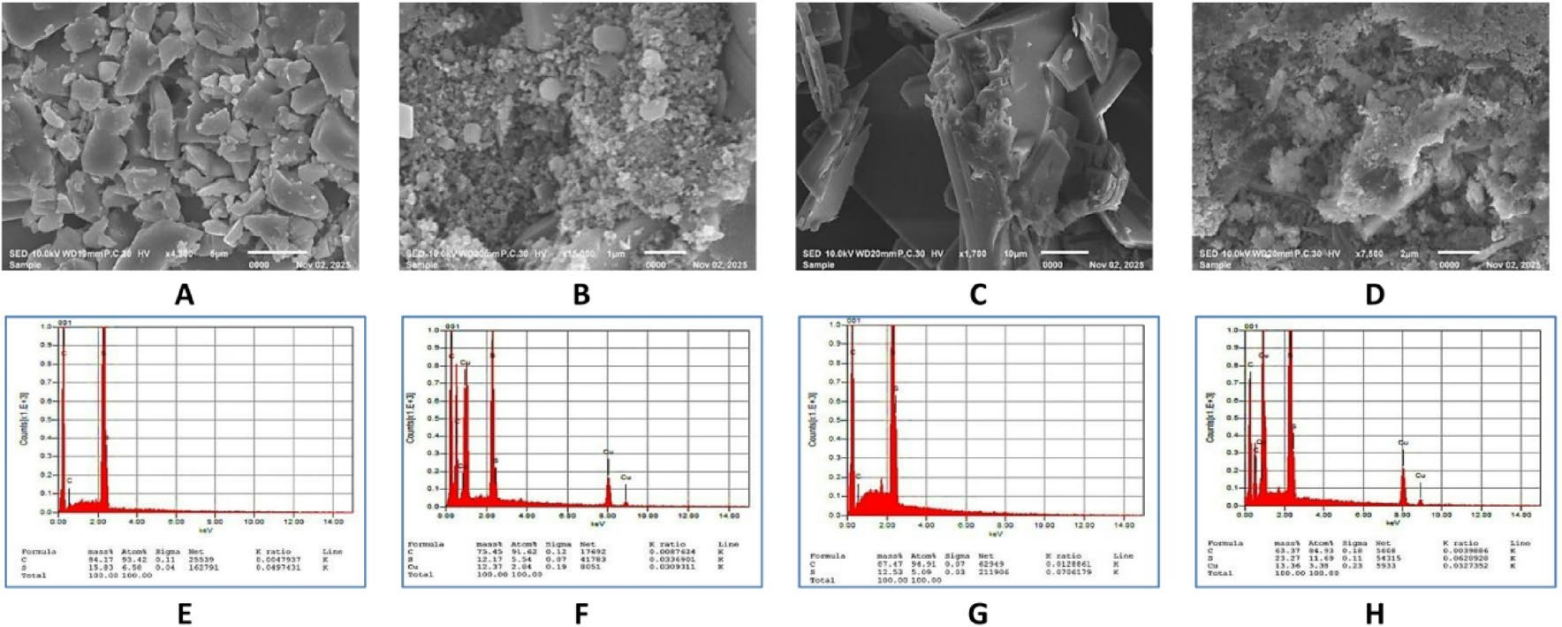
SEM images and corresponding EDS spectra of free ligand S_4_H_2_ (**A**,**E**), binuclear [CuS_4_]_2_ (**B**,**F**), free ligand tBuS_4_H_2_ (**C**,**G**), and mononuclear [CutBuS_4_] (D and H). Scale bars: 5 μm (**A**,**B)**, 1 μm (**C**,**D**).

Scanning electron microscopy (SEM) combined with energy-dispersive X-ray (EDX) spectroscopy provides additional evidence for the morphological consequences of steric control over copper nuclearity (Fig. 2). The free ligands $\:{S}_{4}{H}_{2}$ and $\:tBu{S}_{4}{H}_{2}$ display irregular, aggregated morphologies with particle sizes in the micrometer range (several to tens of µm), consistent with the large hydrodynamic diameters observed in DLS measurements^53,54^. Upon copper coordination, significant changes in particle size and shape are evident. The binuclear $\:[Cu{S}_{4}{]}_{2}$ forms irregular platelet-like structures of approximately 500–800 nm, while the mononuclear $\:\left[Cu\right(tBu{S}_{4}\left)\right]$ exhibits markedly smaller, more uniform spherical particles (100–300 nm) with smoother surfaces. This reduction in size and improved uniformity for $\:\left[Cu\right(tBu{S}_{4}\left)\right]$ aligns with its high absolute zeta potential (− 35.5 mV) and enhanced steric shielding by the eight tert-butyl groups^53,54^. EDX spectra confirm the expected elemental composition. The free ligands show only C and S signals (with minor O from surface oxidation). Both copper complexes exhibit prominent Cu peaks alongside C and S, with approximate atomic ratios Cu: S ≈ 1:4, validating the proposed stoichiometries and successful metal coordination^55^. No impurities or unexpected elements were detected. These SEM/EDX results complement the solution-phase DLS/zeta potential data, demonstrating how remote steric bulk in the $\:tBu{S}_{4}^{2-}$ ligand not only controls nuclearity but also profoundly influences solid-state particle morphology and dispersity^56,57^.

**Table 1:** Hydrodynamic diameter, polydispersity index (PDI), and zeta potential of ligands and complexes in methanol at 25 °C.

Nanomaterials	Zeta potential values (mV) ± SD	Mean diameter (nm)	Polydispersity
S_4_-H_2_	− 26.9 ± 2.6	595	0.436
[CuS_4_]_**2**_	− 18.4 ± 1.7	478	0.364
tBuS_4_-H_2_	− 1.4 ± 0.2	556	0.336
[CutBuS_4_]	− 35.5 ± 1.3	125	0.267

### Redox properties and nuclearity effects

**Fig. 3:**
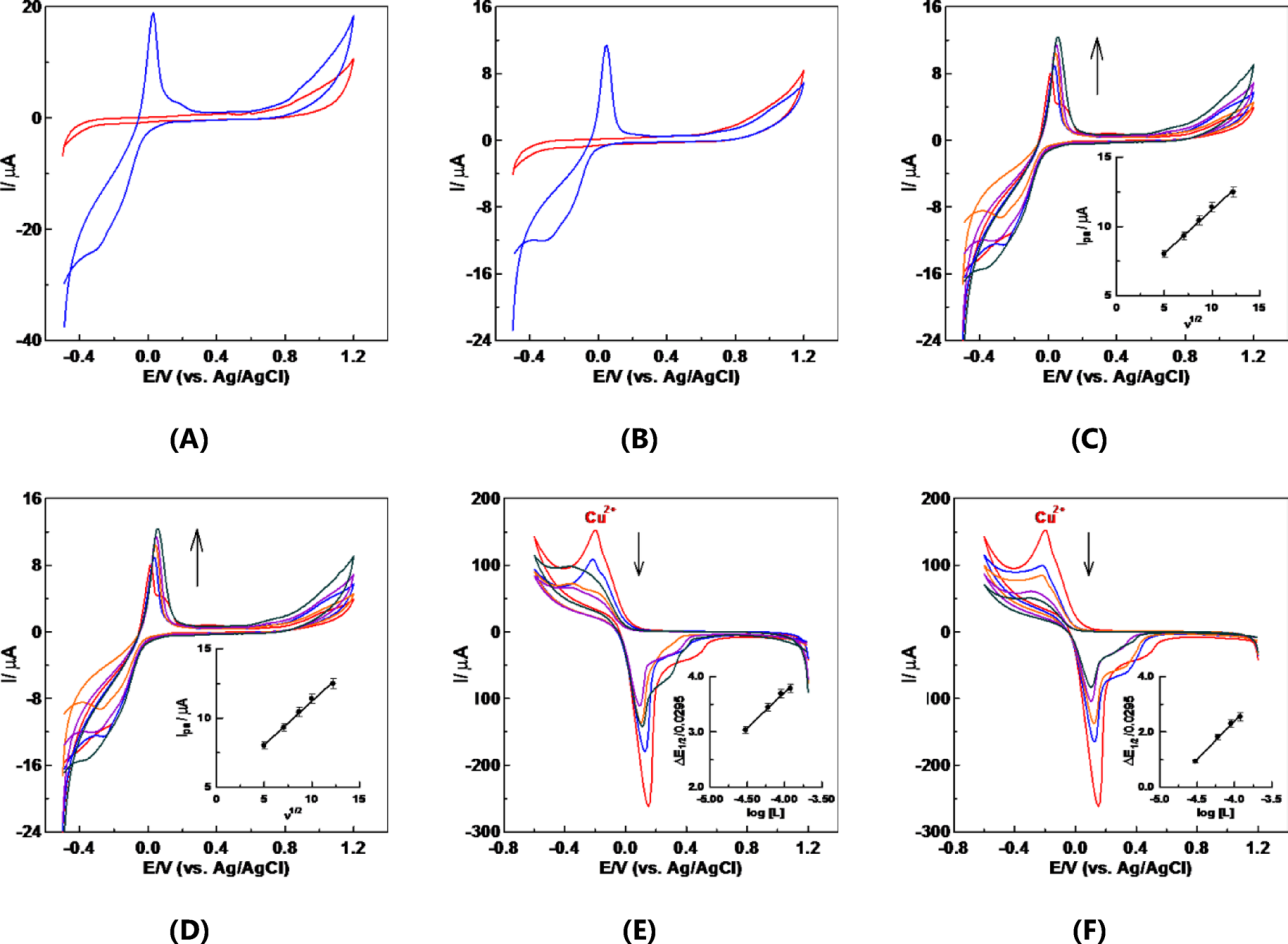
(**A**,**B**) Cyclic voltammograms of 1 × 10^−^⁴ M S_4_^2−^ and tBuS_4_^2−^ ligands (red curves) compared to their copper complexes [CuS_4_]_2_ and [CutBuS_4_] (blue curves) in acetate buffer (pH 5.0) at 100 mV s^−1^. (**C**,**D**) Anodic cyclic voltammograms of 1 × 10^−^⁴ M [CuS_4_]_2_ and [CutBuS_4_] at different scan rates (25–150 mV s^−1^). Insets show I_pa vs. ν^1^/² dependence. (**E**,**F**) Cathodic CV titration of 1.5 × 10^−^⁴ M Cu^II^ with increasing concentrations of S_4_^2−^ and tBuS_4_^2−^ (30–120 µM). Insets show ΔE_½_/0.0295 vs. log [L] plots.

Cyclic voltammetry (CV) reveals distinct redox signatures for the two copper systems that reflect their different nuclearities. The free ligands $\:{S}_{4}^{2-}$ and $\:tBu{S}_{4}^{2-}$ show no redox activity in the window from − 0.5 V to + 1.2 V at 100 mV s^−1^,, whereas both copper complexes $\:[Cu{S}_{4}{]}_{2}$ and $\:\left[Cu\right(tBu{S}_{4}\left)\right]$ exhibit well-defined quasi-reversible $\:{Cu}^{II}$/$\:{Cu}^{I}$ couples with peak-to-peak separations ($\:{\Delta\:}{E}_{p}$) significantly larger than the Nernstian value (59/n mV), consistent with slow electron transfer kinetics or ligand reorganization upon reduction (Fig. 3). Scan rate studies (25–150 mV $\:{s}^{-1}$) confirm quasi-reversible behavior for both systems (Fig. 3C, D; Table 2). As the scan rate increases, $\:{\Delta\:}{E}_{p}$ widens and the $\:{I}_{pc}/{I}_{pa}$ ratio deviates from unity. The anodic peak current ($\:{I}_{pa}$) shows linear dependence on the square root of the scan rate (Randles–Ševčík behavior), indicating diffusion-controlled mass transport^58^. The broadly similar voltammograms observed for [CuS_4_]_2_ and [CutBuS_4_] can be rationalized by their comparable local coordination environments. Although the complexes differ in nuclearity, both contain Cu(II) centres in similar sulfur-rich coordination spheres, which leads to closely related Cu(II)/Cu(I) formal potentials. Under the present conditions, the dicopper core in [CuS_4_]_2_ appears only weakly electronically coupled, so each Cu site behaves largely as an independent redox centre. Consequently, the voltammetric signatures of the binuclear complex resemble those of the mononuclear analogue, indicating that nuclearity mainly affects substrate binding and multi-electron reactivity rather than significantly shifting the intrinsic Cu redox potential.

Cathodic CV titrations provide quantitative insight into solution-phase stability and stoichiometry. Progressive addition of ligand (30–120 µM) to $\:{Cu}^{II}$ (1.5 ×$\:\:{10}^{-4}$ M) decreases the free $\:{Cu}^{II}$ peak current with a cathodic shift, consistent with complex formation (Fig. 3E, F). Analysis of the half-wave potential shift ($\:{\Delta\:}{E}_{1/2}$) using the simplified Lingane Eq. 3 yields slopes near unity when plotting $\:{\Delta\:}{E}_{1/2}$/0.0295 vs. log[L] (1.281 for$\:[Cu{S}_{4}{]}_{2}$ and 1.322 for $\:\left[Cu\right(tBu{S}_{4}\left)\right]$), confirming 1:1 metal-to-ligand stoichiometry in excellent agreement with Job’s method^59^. The calculated formation constants are log $\:{K}_{f}$ = 8.83 for $\:[Cu{S}_{4}{]}_{2}$ and 13.18 for $\:\left[Cu\right(tBu{S}_{4}\left)\right]$. The significantly higher stability of the mononuclear tert-butyl-substituted complex (log $\:{K}_{f}$ = 13.18 vs. 8.83) is particularly notable. Despite greater steric demand, the electron-donating tert-butyl groups strengthen Cu–S interactions, stabilizing the mononuclear species relative to the more flexible binuclear assembly. This enhanced thermodynamic stability provides direct electrochemical evidence that remote steric modification favors discrete mononuclear coordination over higher nuclearity structures.

1 $$\:\varDelta\:{E}_{{1/2}}=\frac{RT}{nF}\left(\mathrm{ln}{K}_{f}+x\:\mathrm{l}\mathrm{n}\left[L\right]\right)$$

2 $$\:\frac{\varDelta\:{E}_{{1/2}}}{\left(RT/nF\right)}=\mathrm{ln}{K}_{f}+x\:\mathrm{l}\mathrm{n}\left[L\right]$$

where $\:R$ = 8.3145 J $\:{mol}^{-1}$ $\:{K}^{-1}$, $\:T$ is absolute temperature, $\:n$ = 2 ($\:{Cu}^{II}$/$\:{Cu}^{I}$), $\:F$ = 96,485 C $\:{mol}^{-1}$, and $\:x$ is coordination number. The Lingane equation can therefore be simplified as follows:

3 $$\:\frac{\varDelta\:{E}_{{1/2}}}{0.0295}=\mathrm{log}{K}_{f}+x\:\mathrm{l}\mathrm{o}\mathrm{g}\left[L\right]$$

**Table 2:** Cyclic voltammetric parameters of [CuS_4_]_2_ and [CutBuS_4_] at different scan rates (25–150 mV s^−1^) in acetate buffer (pH 5.0).

Scan rate (mVs^− 1^)	[CuS_4_]_2_				ΔE_*p*_ (mV)	I_pc_/I_*p*.a._	[CutBuS_4_]				ΔE_*p*_ (mV)	I_pc_/I_*p*.a._
	E_*p*.a._ (V)	I_*p*.a._ (µA)	E_pc_ (V)	I_pc_ (µA)			E_*p*.a._ (V)	I_*p*.a._ (µA)	E_pc_ (V)	I_pc_ (µA)		
25	0.016	8.012	–0.226	11.30	242	1.410	0.025	10.68	–0.244	16.61	269	1.555
50	0.034	9.310	–0.253	12.60	287	1.353	0.028	13.32	–0.253	17.92	281	1.345
75	0.044	10.44	–0.276	9.272	320	0.888	0.030	15.77	–0.304	22.91	334	1.453
100	0.048	11.38	–0.317	12.01	365	1.055	0.035	18.84	–0.322	18.81	357	0.998
150	0.057	12.47	–0.381	15.47	438	1.241	0.039	21.43	–0.349	26.50	388	1.237

### Density functional theory (DFT) calculations: steric control of nuclearity

#### Optimized geometries and coordination environment

**Fig. 4:**
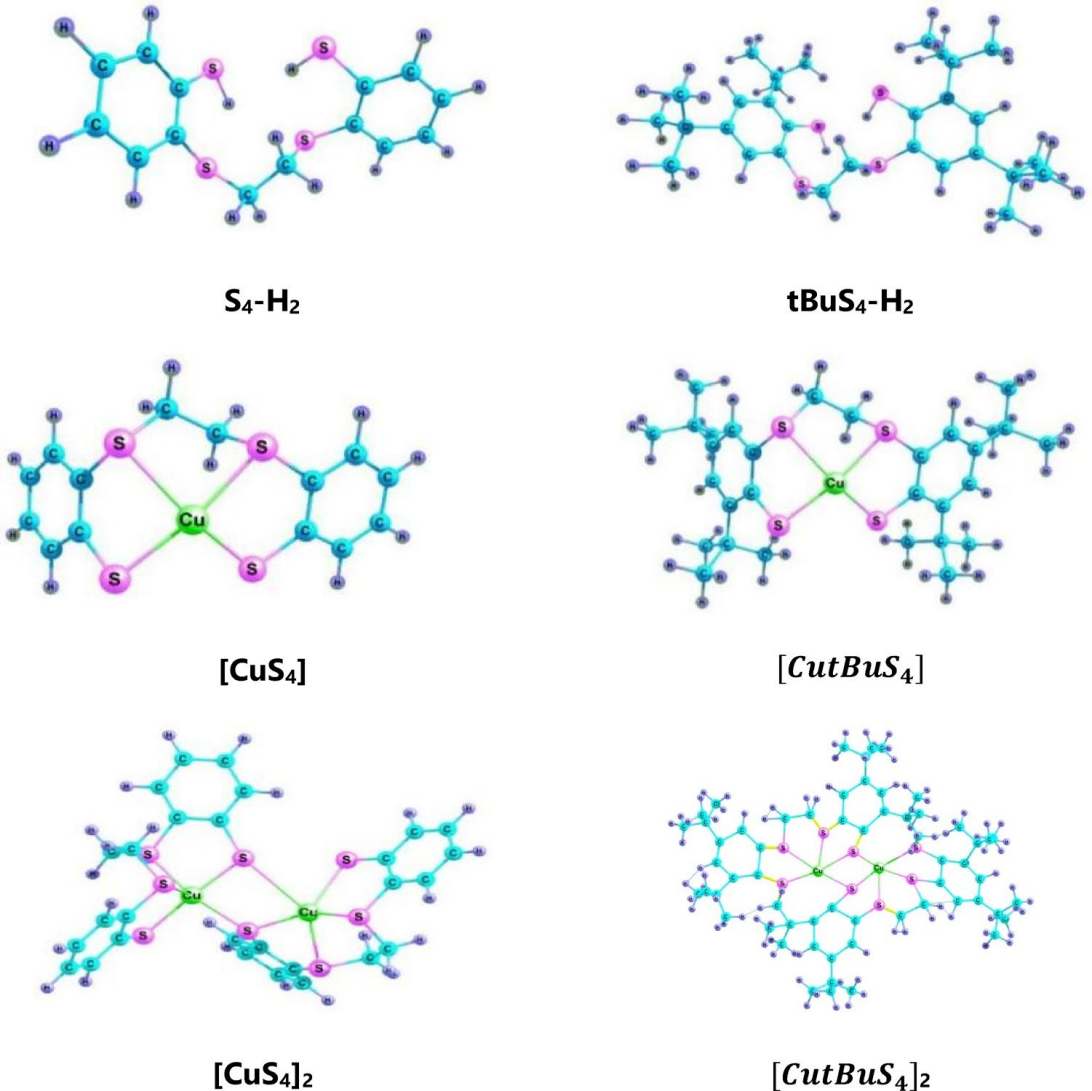
Optimized geometries (PBE1PBE/def2-SVP) of free ligands S_4_^2−^ and tBuS_4_^2−^, mononuclear complexes $\:[Cu{S}_{4}{]}_{2}$ and $\:[Cu{tBuS}_{4}$], and binuclear complexes [CuS_4_]_2_ and [CutBuS_4_]_2_ (hypothetical for tBu case shown for comparison).

Density functional theory calculations were performed at the PBE1PBE/def2-SVP level to provide a comprehensive thermodynamic and electronic rationale for the experimentally observed nuclearity switch from binuclear $\:[Cu{S}_{4}{]}_{2}$ to mononuclear $\:\left[Cu\right(tBu{S}_{4}\left)\right]$ upon remote tert-butyl substitution. Geometry optimizations and frequency analyses confirmed all structures as true minima (no imaginary frequencies). All optimized geometries were confirmed as minima by the absence of imaginary frequencies, and the corresponding Cartesian coordinates are provided in Table S1 (Supporting Information). The optimized geometries (Fig. 4) reveal realistic Cu–S bond lengths ranging from 2.24 to 2.45 Å across both mononuclear complexes, characteristic of covalent $\:{Cu}^{II}$-thiolate interactions with significant d-orbital participation. The tert-butyl-substituted mononuclear complex exhibits systematically shorter average Cu–S distances (by ~ 0.02–0.04 Å) compared to the unsubstituted mononuclear model, consistent with minor geometric compression imposed by the peripheral steric bulk. C–S bond lengths remain in the single-bond regime (1.75–1.84 Å) with modest elongation upon metal coordination, while bite angles around copper (83–97°) deviate substantially from ideal square-planar geometry (Table 3). Nonplanar coordination spheres with significant dihedral distortions further indicate pseudo-tetrahedral to square-pyramidal environments shaped by ligand conformational flexibility and steric interactions.

**Table 3:** Selected optimized bond lengths (Å) and angles (°) for ligands and complexes (PBE1PBE/def2-SVP).

Parameter	S_4_^2−^	tBuS_4_^2−^	[CuS_4_]_2_	[CutBuS_4_]
Cu1–S1	–	–	2.449	2.415
Cu1–S2	–	–	2.383	2.345
Cu1–S3	–	–	2.260	2.259
Cu1–S4	–	–	2.252	2.244
S1–C (avg)	1.824	1.824	1.837	1.836
S3–C (avg)	1.762	1.774	1.752	1.769
S1–Cu1–S2 (°)	–	–	83.6	86.0
S3–Cu1–S4 (°)	–	–	95.6	96.8

#### Thermodynamics of nuclearity

The defining thermodynamic insight emerges from dimerization free energy calculations (Eq. 4):

4 $$\:{\Delta\:}{G}_{dimer}=G\left(\left[C{u}_{2}\left(L\right)\right]\right)-2G\left(\left[Cu\left(L\right)\right]\right)$$

For the unsubstituted $\:{S}_{4}^{2-}$ system, $\:{\Delta\:}{G}_{dimer}\approx\:-2.6$ kcal $\:{mol}^{-1}$ renders binuclear formation thermodynamically favorable, consistent with the experimental isolation of $\:[Cu{S}_{4}{]}_{2}$. In marked contrast, the tert-butyl-substituted $\:tBu{S}_{4}^{2-}$ system yields $\:{\Delta\:}{G}_{dimer}>0$ due to severe enthalpic penalties from steric clashes between opposing tert-butyl groups in the hypothetical binuclear structure (Table 4). This steric repulsion restricts vibrational and rotational entropy, producing a substantial free energy barrier to dimerization. The computed thermodynamic switch, negative $\:{\Delta\:}{G}_{dimer}$ for the flexible ligand versus positive for the sterically encumbered ligand, provides unambiguous computational validation of the design principle that remote steric modification can selectively disfavor higher nuclearity assemblies.

**Table 4:** Key thermodynamic parameters (PBE1PBE/def2-SVP, au unless noted).

Structure	ZPVE	G	E (kcal/mol)	S (cal/mol K)	Cv (cal/mol K)
S_4_H_2_	−2132.370	−2132.419	162.2	142.1	67.7
tBuS_4_H_2_	−2759.709	−2759.783	457.9	239.9	155.4
[CuS_4_]	−3771.303	−3771.352	152.3	142.5	68.9
[CutBuS_4_]	−4398.644	−4398.717	447.8	240.9	156.9
[CuS_4_]_2_	−7542.623	−7542.699	306.6	241.2	143.2
[CutBuS_4_]_2_	−8798.632	−8798.640	865.2	280.5	248.6

#### Spin-state energetics of [CuS_4_]_2_

For [CuS_4_]_2_, both spin states were optimized at the PBE1PBE/def2-SVP level. The triplet state (E = − 7542.698864 a.u.) lies 18.26 kcal mol^−1^ lower in energy than the broken-symmetry singlet (E = − 7542.669764 a.u.), giving Δ*E*_S–T_ = − 18.26 kcal mol^−1^. This establishes the triplet as the ground state, and all structural, thermodynamic and electronic analyses for [CuS_4_]_2_ were therefore based on the triplet-state geometries. The energetic preference for the triplet state indicates a ferromagnetically favored or very weakly antiferromagnetically coupled dicopper core, highlighting the importance of identifying the correct spin ground state when interpreting the electronic structure and catalytic behaviour of these complexes.

#### Frontier orbitals and reactivity descriptors

**Fig. 5:**
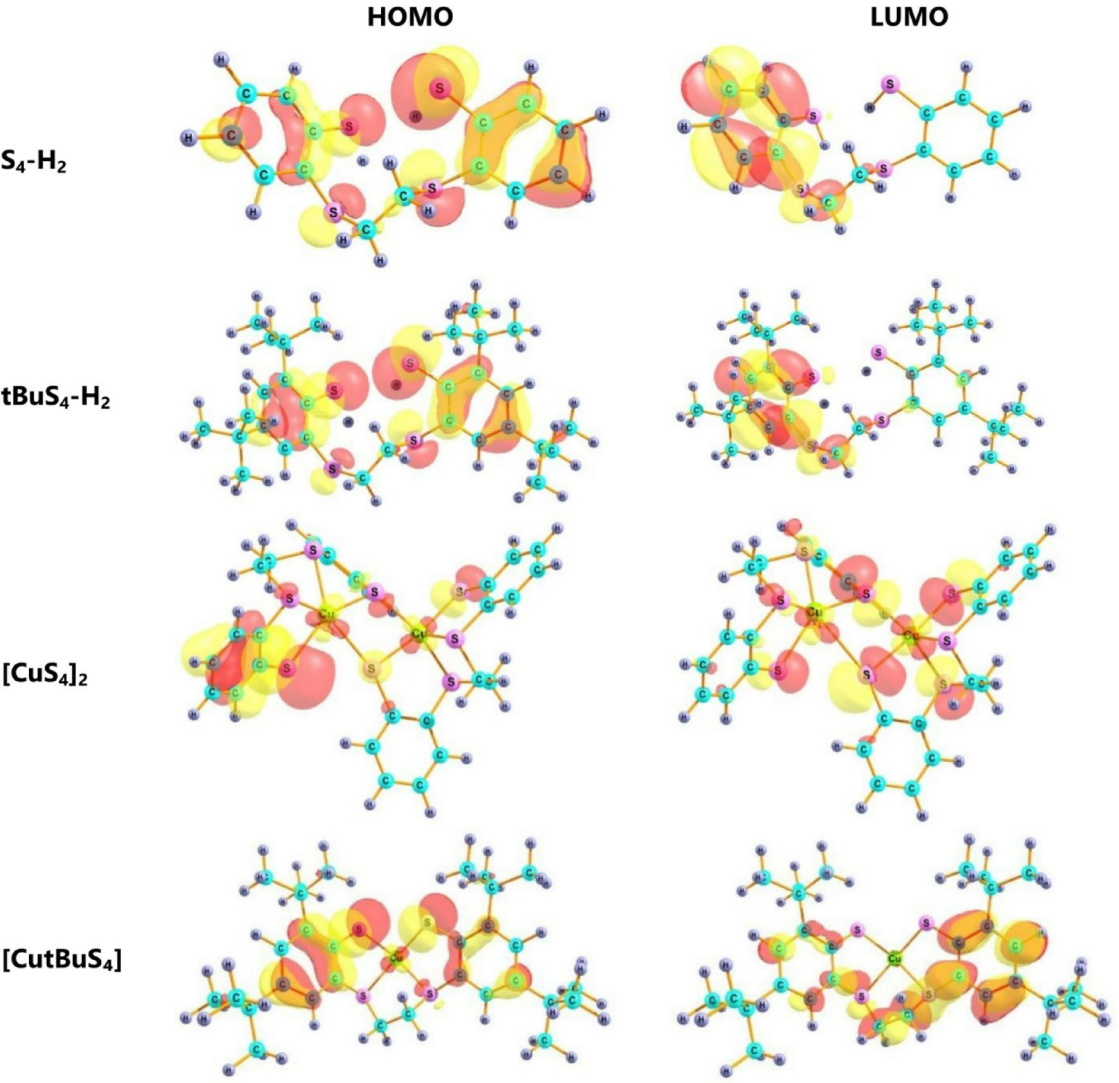
Contour plots of the α-spin HOMO (left) and LUMO (right) for S_4_H_2_, tBuS_4_H_2_, $\:[Cu{S}_{4}{]}_{2}$, and $\:[Cu{tBuS}_{4}$] (isovalue = 0.03 a.u.). The corresponding β-spin orbitals display analogous spatial distributions and are omitted for clarity.

Frontier molecular orbital (FMO) analysis elucidates the electronic consequences of this structural divergence. Free ligands exhibit large HOMO–LUMO gaps (~ 5.3 eV), reflecting high kinetic stability and low inherent reactivity. The inductive electron donation from tert-butyl groups slightly elevates the HOMO energy, modestly narrowing the gap in$\:\:tBu{S}_{4}{H}_{2}\:$^60,61^. Copper coordination dramatically reduces $\:{\Delta\:}{E}_{gap}$, most pronounced for the mononuclear $\:\left[Cu\right(tBu{S}_{4}\left)\right]$ (1.66 eV) due to extensive Cu–S σ- and π-orbital mixing that enhances electronic delocalization (Fig. 5; Table 5). Global reactivity descriptors derived via Koopmans’ theorem^62^ reveal stark nuclearity-dependent trends: the mononuclear complex displays the lowest chemical hardness ($\:\eta\:=0.83$ eV), highest molecular softness ($\:S=1.21$ $\:{eV}^{-1}$), greatest electrophilicity ($\:\omega\:=11.97$ eV), and enhanced nucleophilicity ($\:N=5.38$ eV). These metrics indicate greater intrinsic reactivity and polarizability for the sterically isolated mononuclear species compared to the binuclear complex, where cooperative charge delocalization across metal centers moderates these properties.

**Table 5:** Frontier orbital energies and global reactivity descriptors for the ligands and copper complexes (PBE1PBE/def2-SVP).

Compound	E_HOMO_ (eV)	E_LUMO_ (eV)	ΔE_gap_ (eV)	η (eV)	S (eV^−1^)	χ (eV)	ω (eV)	*N* (eV)
S_4_H_2_	–6.178	–0.851	5.327	2.664	0.375	3.515	2.319	1.319
tBuS_4_H_2_	–5.941	–0.655	5.286	2.643	0.378	3.298	2.058	1.248
$\:[Cu{S}_{4}{]}_{2}$	–5.431	–0.893	4.538	2.269	0.441	3.162	2.203	1.393
$\:[Cu{tBuS}_{4}$]	–5.280	–3.625	1.655	0.828	1.208	4.453	11.97	5.379

Spatial FMO distributions provide further insight into reactivity differences (Fig. 5). In free ligands, HOMOs localize primarily on thiolate sulfur lone pairs with aromatic π-contributions, while LUMOs reside in C–S antibonding orbitals. Coordination fundamentally reorganizes this picture: complex HOMOs gain substantial Cu d-orbital character hybridized with thiolate p-orbitals, while LUMOs become predominantly metal-centered with strong thiolate antibonding contributions. The mononuclear $\:\left[CutBu{S}_{4}\right]$ exhibits particularly low-energy LUMOs dominated by Cu$\:{d}_{xz/yz}$ character, explaining its superior electron-accepting ability and correlating with the experimentally observed stronger initial substrate binding.

#### Molecular electrostatic potential (MEP) surfaces

**Fig. 6:**
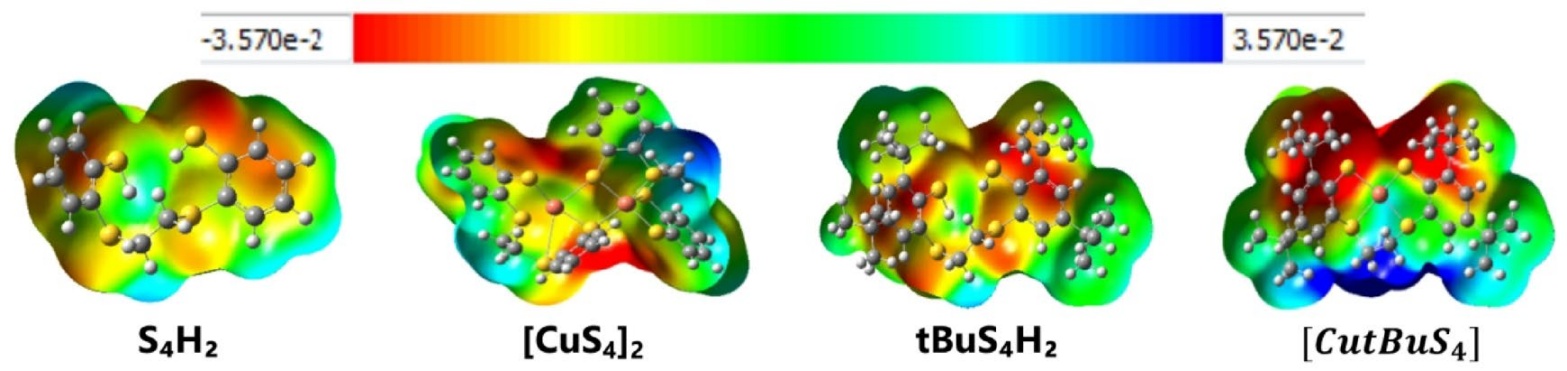
MEP maps (isovalue = 0.002 au), color scale: red (negative), green (neutral), blue (positive).

Molecular electrostatic potential (MEP) surfaces were analyzed to visualize the computed charge distribution and its implications for coordination and reactivity^63^. The free ligands S_4_H_2_ and tBuS_4_H_2_ display strongly negative potentials (red regions) at the deprotonated thiolate sulfur sites and adjacent aromatic rings, identifying these positions as prime loci for electrophilic metal coordination, while positive potentials (blue) are concentrated at aromatic C–H and methylene groups. The tert-butyl substituents slightly disperse electron density from the sulfur donors, yielding marginally less negative potentials around the tBu-substituted thiolates. Upon coordination, both complexes retain pronounced negative character at the Cu-bound thiolate donors while moderate positive potential appears at the copper centres. In the mononuclear [CutBuS_4_] complex, this negative charge is more localized around a single CuS_4_ core, whereas in binuclear [CuS_4_]_2_ the negative potential is delocalised over the bridging sulfurs and aromatic ligands and the dicopper core shows interspersed neutral/positive regions indicative of charge sharing and orbital overlap (Fig. 6). These electrostatic profiles rationalise the nuclearity-dependent substrate binding behaviour: more localized thiolate nucleophilicity in the mononuclear species versus a more distributed, cooperative electrostatic environment in the binuclear assembly.

#### TD-DFT and simulated UV–Vis spectra

Time-dependent DFT (TD-DFT) calculations validate the experimental UV–Vis assignments. The 20 lowest singlet excitations reproduce observed spectral progressions with Gaussian broadening (FWHM = 0.3 eV): free ligands show intense ligand-centered $\:\pi\:\to\:{\pi\:}^{\mathrm{*}}$ bands (290–295 nm), while coordination induces bathochromic shifts to LMCT-dominated transitions. The mononuclear $\:\left[Cu\right(tBu{S}_{4}\left)\right]$ exhibits a primary LMCT at ~ 300 nm (f = 0.0709), while the binuclear $\:[Cu{S}_{4}{]}_{2}$ shows a more intense, further red-shifted band at ~ 305 nm (f = 0.1997) with greater delocalization across the dicopper unit (Table 6 and Fig. S3 in Supporting Information). These computed intensities and shifts match experimental green coloration and nuclearity-dependent spectral fingerprints^64^. The comprehensive DFT analysis establishes a clear causal chain: remote tert-butyl substitution (1) thermodynamically disfavors binuclear assembly ($\:{\Delta\:}{G}_{dimer}>0$) through steric/enthalpic penalties, (2) electronically activates the mononuclear species via narrowed HOMO–LUMO gap and enhanced softness, (3) preserves localized thiolate nucleophilicity for substrate binding, and (4) produces distinct spectroscopic signatures validated by TD-DFT. This multi-faceted computational evidence provides the rigorous theoretical foundation linking ligand steric design to the observed nuclearity switch and its downstream catalytic consequences, perfectly complementing the experimental structural and redox data. While the precise structural assignment is strongly supported by spectroscopic, electrochemical, and computational evidence, the absence of single-crystal X-ray diffraction data represents a limitation of the present study.

**Table 6:** Key TD-DFT parameters for the most intense bands.

Compound	λ_max_ (nm)	E_ex_ (eV)	f	Dominant transition	Assignment
S_4_H_2_	290	4.28	0.0447	HOMO → LUMO + 4 (π → π*)	LC
tBuS_4_H_2_	295	4.20	0.1336	HOMO → LUMO + 3 (π → π*)	LC
$\:[Cu{S}_{4}{]}_{2}$	305	4.07	0.1997	S → Cu / delocalized CT	LMCT / CT
$\:\left[CutBu{S}_{4}\right]$	300	4.13	0.0709	Ligand/S → Cu	LMCT

LC = ligand-centered; LMCT = ligand-to-metal charge transfer; CT = charge transfer.

### EPR spectroscopy

**Fig. 7:**
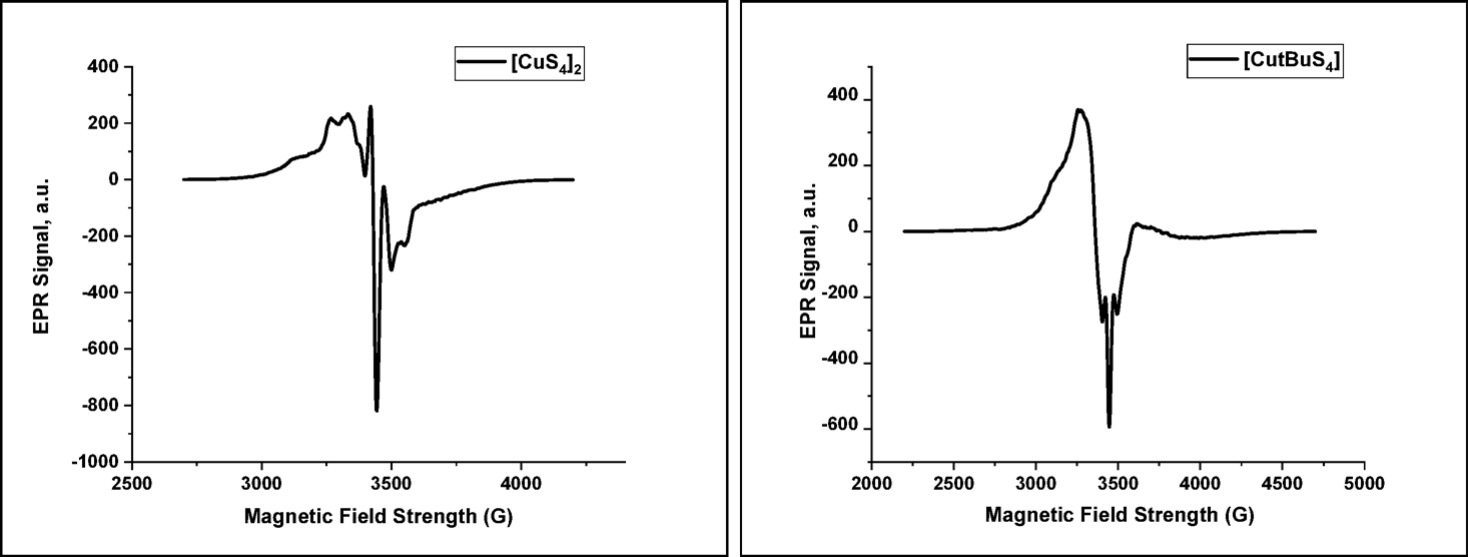
X-band EPR spectra of the binuclear complex [CuS_4_]_2_ and the mononuclear complex [CutBuS_4_] recorded at room temperature, together with the corresponding *g*∥ and *g*⊥ values.

Electron paramagnetic resonance (EPR) spectroscopy was employed to probe the local electronic environment and nuclearity of the Cu(II) centres in [CuS_4_]_2_ and [CutBuS_4_] (Fig. 7). The X-band spectra of both complexes display axial line shapes consistent with Cu(II) ions having predominantly *dx*2 − *y*2 ground states, in line with the well-established correlations between *g*∥, A‖ and tetragonally distorted Cu(II) geometries^65^. For [CuS_4_]_2_, the g-tensor components *g*∥=2.01914 and *g*⊥=2.02607 fall within the range typical of Cu(II) centres in distorted square-pyramidal thiolate environments and support the presence of exchange-coupled dicopper sites. These values are consistent with the pseudo-tetrahedral to square-pyramidal CuS_4_ coordination spheres obtained from the PBE1PBE/def2-SVP optimised structures.

In contrast, [CutBuS_4_] exhibits higher g values, *g*∥ = 2.06008 and *g*⊥ = 2.11384, and a larger Δ*g*, indicative of a more strongly tetragonally elongated and rhombic coordination sphere around a mononuclear Cu(II) center, consistent with trends predicted by DFT studies on tetragonal Cu(II) model complexes^66^. The increased *g*∥ and Δ*g* are in agreement with the shorter Cu–S bonds and enhanced tetragonal distortion revealed by the DFT calculations for the tert-butyl-substituted system. The broader, less resolved features for [CutBuS_4_] are consistent with a distribution of conformations at a single Cu(II) site rather than well-resolved hyperfine structure, whereas the [CuS_4_]_2_ spectrum reflects antiferromagnetically coupled Cu(II) centers with a residual signal from uncoupled or weakly coupled sites. Overall, the EPR parameters corroborate the DFT-derived picture in which the less hindered ligand favours binuclear Cu(II) assemblies with distorted square-pyramidal CuS_4_ units, while the tert-butyl-substituted ligand stabilizes a more anisotropic, tetragonally elongated mononuclear CuS_4_ environment.

### Catechol oxidase activity: nuclearity controls productive substrate binding

**Fig. 8:**
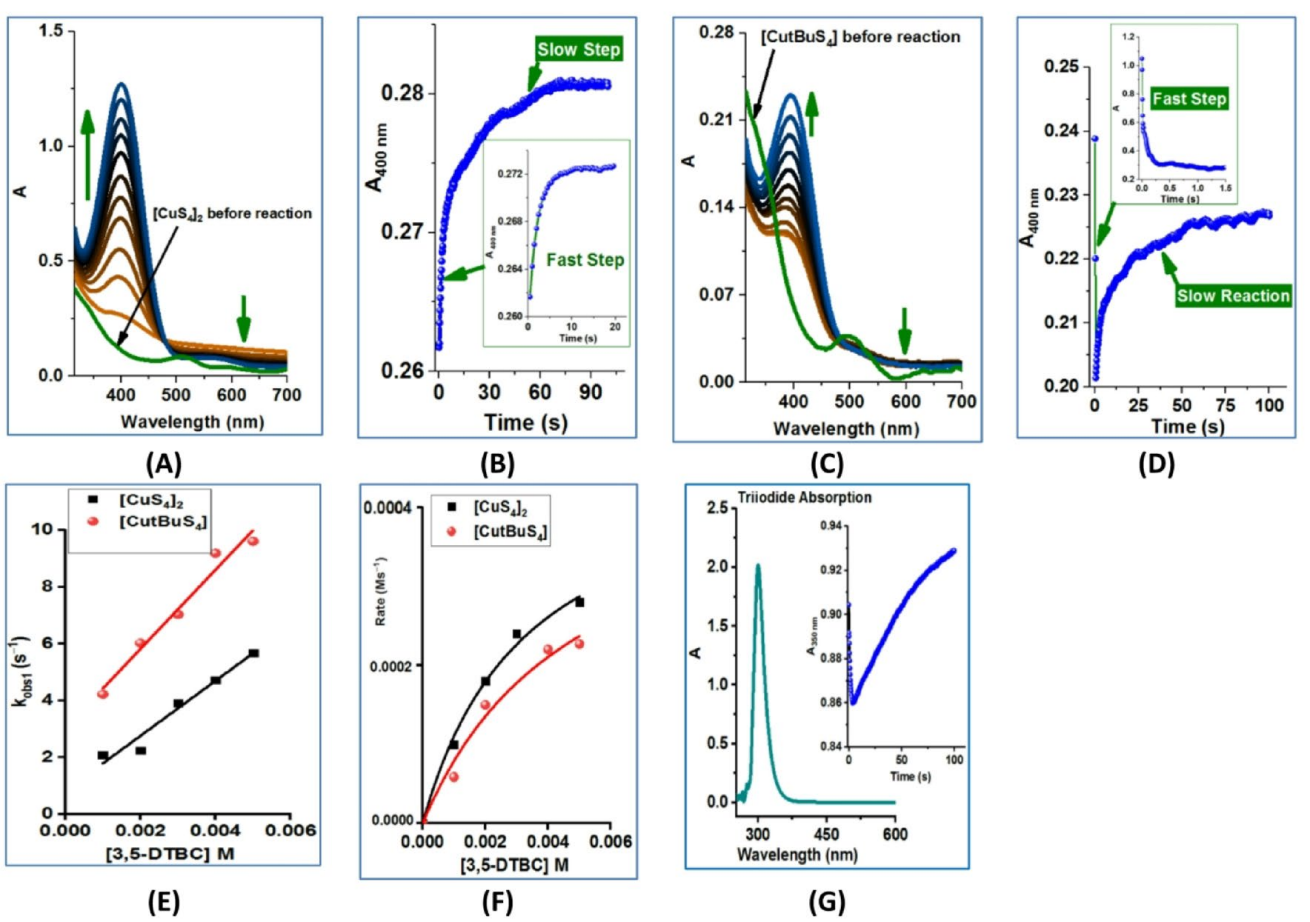
Time-dependent UV–vis spectral changes during aerobic oxidation of 3,5-DTBC (100 equiv) catalyzed by (**A**) binuclear $\:[Cu{S}_{4}{]}_{2}$ and (**C**) mononuclear $\:\left[CutBu{S}_{4}\right]$ in methanol at 25 °C. The band at 400 nm corresponds to 3,5-DTBQ formation; (**B**,**D**) Absorbance–time traces showing fast pre-equilibrium followed by steady-state quinone formation. (**E**) Plot of k_obs_ (fast phase) vs. [3,5-DTBC] for the reversible binding step; (**F**) Michaelis–Menten plot of the catalytic phase; (**G**) detection of hydrogen peroxide during the reduced product of O_2_ during catechol oxidase-like catalysis. Time-dependent UV–vis spectra showing the growth of the triiodide band at λ = 351 nm upon aerobic oxidation of 3,5-DTBC (100 equiv) catalyzed by [CuS_4_]_2_ in methanol at 25 °C in the presence of excess KI and catalytic ammonium molybdate.

The aerobic oxidation of 3,5-di-tert-butylcatechol (3,5-DTBC) to 3,5-di-tert-butyl-1,2-benzoquinone (3,5-DTBQ) was monitored at 400 nm (ε ≈ 1670 $\:{M}^{-1}$ $\:{cm}^{-1}$) in methanol at 25 °C^67^. Electrochemical measurements were performed in aqueous acetate buffer to obtain well‑defined Cu(II)/Cu(I) redox behavior under controlled ionic strength and pH and to maintain electrode stability and reproducible peak shapes, whereas catalytic assays were carried out in methanol to provide adequate solubility and stability for both the complexes and the substrates (3,5‑DTBC and OAP) and to match conditions commonly employed in catechol oxidase and phenoxazinone synthase model studies. Screening assays using 100 equivalents of 3,5-DTBC with 5 × $\:{10}^{-4}$ M complex solutions revealed apparently faster initial quinone formation for the binuclear $\:[Cu{S}_{4}{]}_{2}$ (saturation within ~ 65 s) compared to the mononuclear $\:\left[CutBu{S}_{4}\right]$ (Fig. 7A, C)^67–69^. Control experiments confirmed negligible background oxidation in the absence of catalyst or under anaerobic conditions. topped-flow kinetic studies under pseudo-first-order conditions ([3,5-DTBC] = 0.5–5.0 mM, [complex] = 2.5 × $\:{10}^{-1}$ M) provided the mechanistic resolution, revealing biphasic behavior for both complexes diagnostic of rapid pre-equilibrium substrate binding followed by rate-determining catalytic oxidation (Fig. 8B,D). Global fitting to a two-step sequential model yielded the microscopic rate constants summarized in Table 7 and presented in Fig. 8E,F. The binuclear complex exhibits 2.5-fold higher substrate affinity in the pre-equilibrium binding step ($\:{K}_{a}=1166$ $\:{M}^{-1}$ vs. 464 $\:{M}^{-1}$ for$\:\:\left[CutBu{S}_{4}\right]$), consistent with cooperative bidentate coordination across the dicopper center^11,70–73^. Michaelis–Menten analysis of the steady-state phase reveals virtually identical maximum turnover rates for both complexes ($\:{V}_{max}\approx\:4.8\times\:{10}^{-4}$ M $\:{s}^{-1}$, TOF ≈ 1700 $\:{h}^{-1}$), but the binuclear species achieves saturation at substantially lower substrate concentrations due to its lower $\:{K}_{M}$ (3.4 mM vs. 5.0 mM)^72–74^. Thus nuclearity primarily governs productive substrate binding rather than intrinsic oxidation capability, with the dicopper system’s superior pre-equilibrium coordination translating into enhanced apparent activity under typical screening conditions. Attempts to detect substrate-bound intermediates (catechol/OAP adducts) by ESI-MS were unsuccessful, likely due to their transient nature and instability under ionization conditions. Their involvement is instead supported by kinetic and spectroscopic evidence.

**Table 7:** Kinetic and activity parameters for the aerobic oxidation of 3,5-DTBC catalyzed by [CuS_4_]_2_ and [CutBuS_4_] in methanol at 25 °C.

Complex	Fast coordination step				Slow oxidation step		
	k_1_[M^− 1^ s^− 1^]	k_− 1_ [s^− 1^]	K_a_ [M^− 1^]	K_d1_ M	K_M_ 10^− 3^ M	V_MAX_ 10^− 4^ [Ms^− 1^]	TOF [h^− 1^]
$\:[Cu{S}_{4}{]}_{2}$	968 ± 114	0.83 ± 0.12	1166	0.0085	3.4	4.8	1728
$\:\left[CutBu{S}_{4}\right]$	1392 ± 142	3.0 ± 0.4	464	0.0021	5.0	4.7	1692

### Oxidase-type mechanism confirmed by H_2_O_2_ detection

Hydrogen peroxide quantification using the classical iodide assay provides direct evidence for the oxidase-type mechanism operating in both copper complexes^75,76^. In a typical experiment, 100 equivalents of 3,5-DTBC were added to air-saturated methanolic solutions containing 5 × $\:{10}^{-4}$ M complex, excess KI, and catalytic ammonium molybdate. The reaction was monitored by the growth of the triiodide absorption at 351 nm (ε = 25,700 $\:{M}^{-1}$ $\:{cm}^{-1}$) (Fig. 8G). Both binuclear $\:[Cu{S}_{4}{]}_{2}$ and mononuclear $\:\left[CutBu{S}_{4}\right]$ generated H_2_O_2_ in amounts stoichiometric with 3,5-DTBQ formation, showing no significant nuclearity-dependent differences in oxygen reduction stoichiometry. Control experiments under anaerobic conditions or without catalyst produced negligible triiodide, confirming that H_2_O_2_ formation accompanies aerobic catalytic turnover. These results establish that both complexes operate via true oxidase pathways (O_2_ → 2H_2_O_2_) rather than dioxygenase mechanisms, with individual $\:{Cu}^{II}$-thiolate units fully competent for two-electron oxygen reduction coupled to catechol oxidation. This mechanistic confirmation eliminates ambiguity and validates the direct comparison of nuclearity-dependent binding kinetics established in Sect.  3.4.

### Phenoxazinone synthase activity: nuclearity controls oxidation turnover

**Fig. 9:**
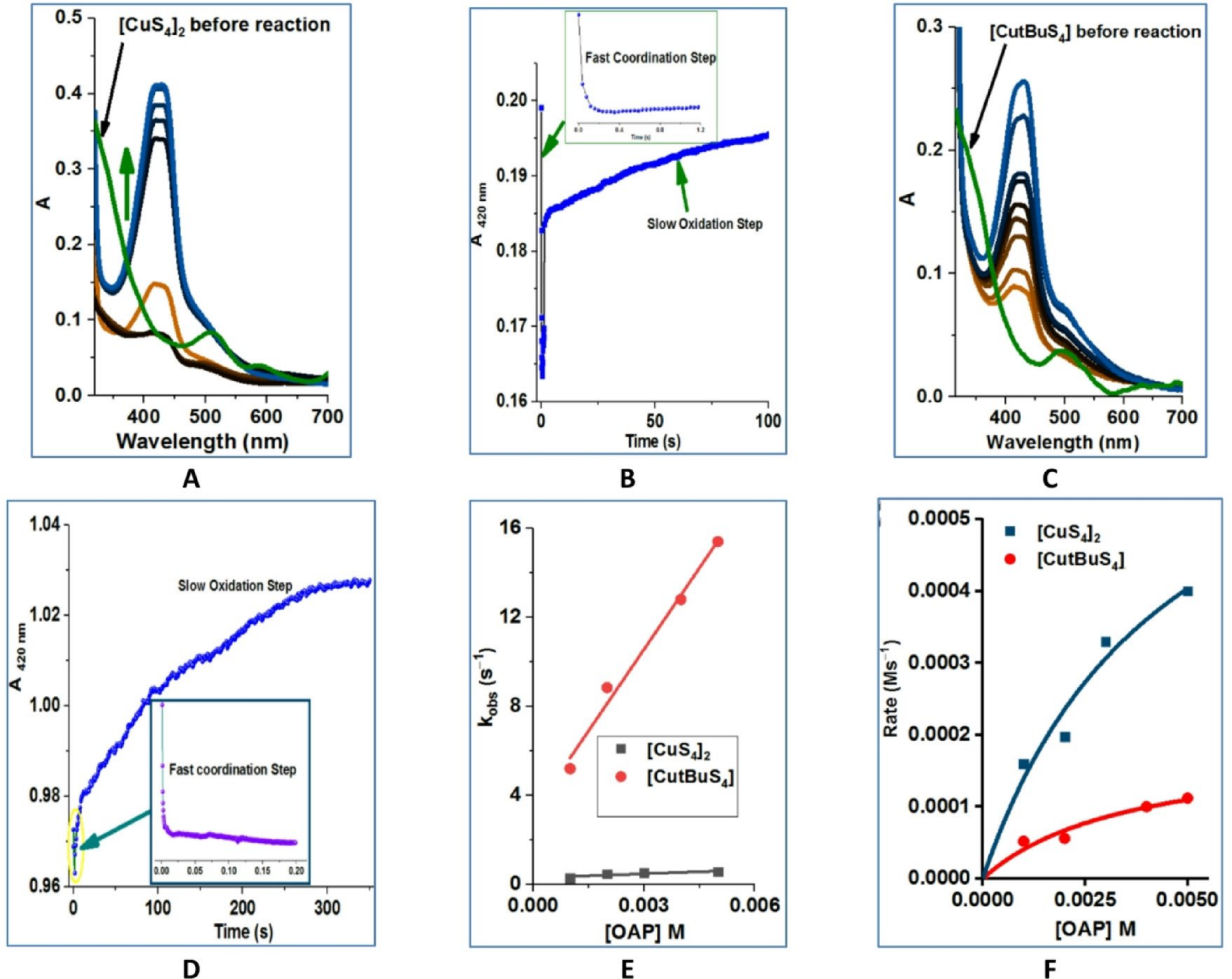
Time-dependent UV–vis spectral changes during aerobic oxidation of OAP (100 equiv) catalyzed by (**A**) binuclear $\:[Cu{S}_{4}{]}_{2}$ and (**C**) mononuclear $\:\left[CutBu{S}_{4}\right]\:$in methanol at 25 °C. The band at 430 nm corresponds to APX formation; (**B**,**D**) Time-resolved spectra and absorbance–time traces at 430 nm; Kinetic analysis of OAP oxidation. (**E**) Plot of the fast-phase observed rate constant vs. [OAP]; (**F**) Michaelis–Menten plot of the steady-state phase.

The aerobic oxidative coupling of o-aminophenol (OAP) to 2-aminophenoxazin-3-one (APX) was monitored by the characteristic APX absorption band at ~ 430 nm in methanol at 25 °C (ε ≈ 1.6 × 10⁴ M^−1^ cm^−1^)^77, 78,79^. Stopped-flow kinetic studies under pseudo-first-order conditions ([OAP] = 0.5–5.0 mM, [complex] = 2.5 × $\:{10}^{-4}$ M) revealed the characteristic two-step profile for both complexes: rapid pre-equilibrium substrate binding followed by slower catalytic turnover (Fig. 9A-D). The corresponding microscopic rate constants are summarized in Table 8.

A striking binding–turnover inversion distinguishes the two systems. The mononuclear $\:\left[CutBu{S}_{4}\right]$ exhibits 3.6-fold stronger OAP binding in the pre-equilibrium step (K_a_ = 749 M^−1^ vs. 205 M^−1^ for $\:[Cu{S}_{4}{]}_{2}$),, consistent with greater steric accessibility at its single copper center [69,72]. However, Michaelis–Menten analysis of the catalytic phase reveals the opposite trend, with the binuclear complex achieving a significantly higher maximum velocity (V_max_ = 7.7 × 10^−^⁴ M s^−1^, TOF = 2772 h^−1^) compared to the mononuclear species (V_max_ = 1.9 × 10^−4^ M s^−1^, TOF = 684 h^−1^), despite comparable K_M_ values. This nuclearity-dependent reversal provides the mechanistic climax of the study. While catechol oxidation (Sect.  3.5) is primarily binding-limited, favoring the binuclear system through more effective substrate coordination, the six-electron oxidative coupling of OAP requires efficient O_2_ activation and multi-electron transfer during the rate-determining step. In this regime, the dicopper complex excels through cooperative activation of dioxygen and concerted oxidation at the bimetallic site, whereas the mononuclear complex—despite favorable substrate binding—cannot effectively support this multi-electron process, establishing $\:[Cu{S}_{4}{]}_{2}$), as the superior phenoxazinone synthase mimic^74^.

**Table 8:** Kinetic and activity parameters for the aerobic oxidation of OAP catalyzed by [CuS_4_]_2_ and [CutBuS_4_] in methanol at 25 °C.

Complex	Fast coordination step				Slow oxidation step		
	k_1_[M^− 1^ s^− 1^]	k_− 1_ [s^− 1^]	K_a_ [M^− 1^]	K_d1_ M	K_M_ 10^− 3^ M	V_MAX_ 10^− 4^ [Ms^− 1^]	TOF [h^− 1^]
$\:[Cu{S}_{4}{]}_{2}$	610.4 ± 32	3.30 ± 0.10	205	0.0048	4.5	7.7	2772
$\:\left[CutBu{S}_{4}\right]$	2436.0 ± 0.197	3.25 ± 0.67	749	0.0013	3.7	1.9	684

### 4-Nitrocatechol binding without oxidation: coordination ≠ turnover

**Fig. 10:**
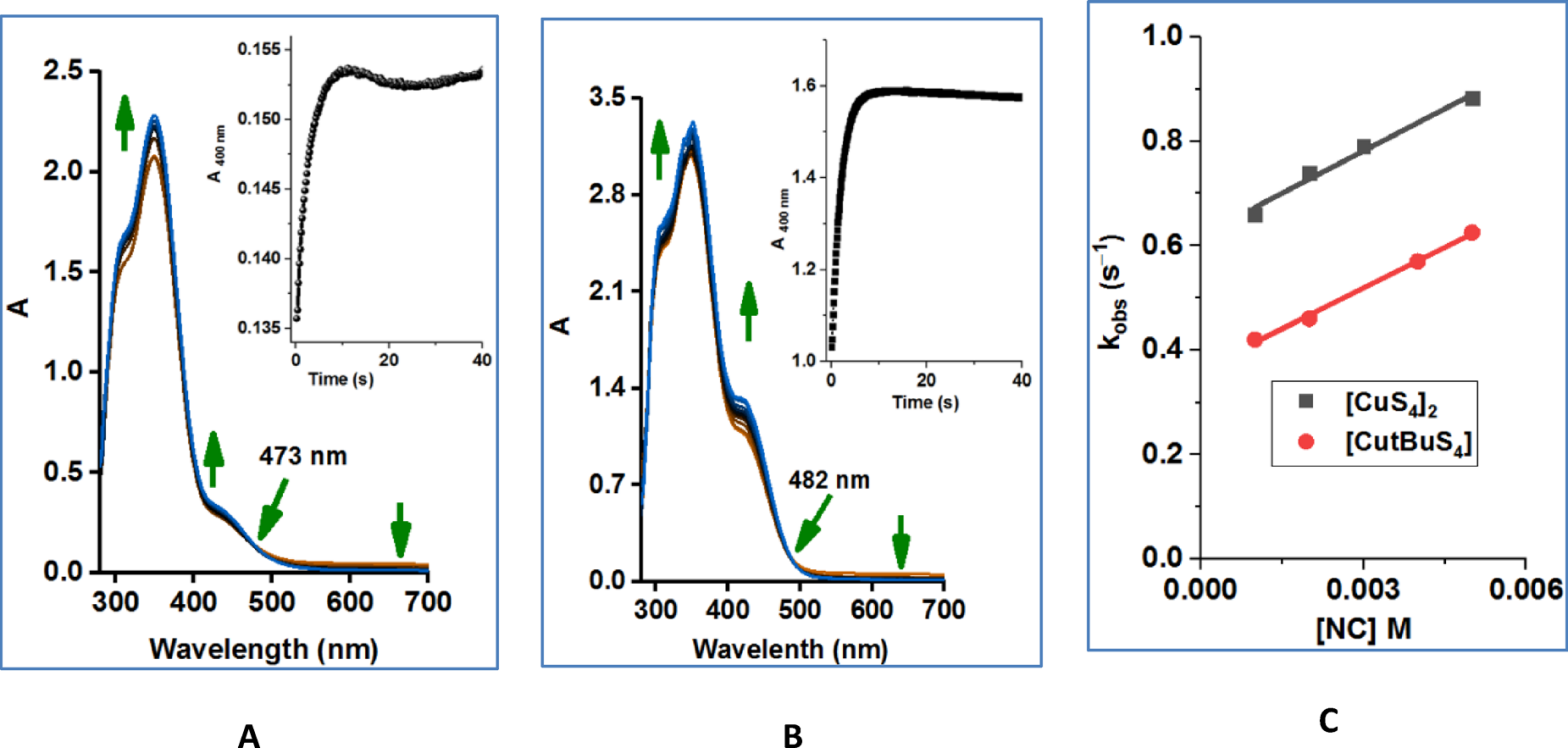
Time-dependent UV–vis spectral changes upon addition of NC (100 equiv) to (**A**) $\:[Cu{S}_{4}{]}_{2}$ and (**B**) $\:\left[CutBu{S}_{4}\right]$ in methanol at 25 °C, showing clean isobestic points at 473 nm and 482 nm, respectively. Insets: absorbance–time traces. (**C**) Plot of k_obs_ vs. [NC].

The interaction of both complexes with 4-nitrocatechol (NC) demonstrates that substrate binding occurs readily but requires additional factors beyond coordination for catalytic turnover. Addition of excess NC (100 equiv) to methanolic solutions produced rapid spectral changes characterized by intense catecholate-to-$\:{Cu}^{II}$ LMCT bands at 520–550 nm and bleaching of the original d-d transitions, maintaining sharp isosbestic points at 473 nm for $\:[Cu{S}_{4}{]}_{2}$ and 482 nm for $\:\left[Cu\right(tBu{S}_{4}\left)\right]$ (Fig. 10A, B). No quinone formation was detected in the 400–420 nm region even after 24 h, despite clean adduct formation confirmed by single-exponential stopped-flow kinetics (Table 9)^79^. The mononuclear complex again showed superior binding affinity ($\:{K}_{a}=144\:{M}^{-1}$ vs. 86.9 $\:{M}^{-1}$ for the binuclear species), consistent with greater steric accessibility of its single copper site^49,80^. This strong NC coordination without subsequent oxidation provides the definitive mechanistic control: substrate binding alone cannot drive catalysis in these sulfur-ligated copper systems. Efficient turnover therefore demands both productive coordination and favorable thermodynamics for electron transfer from substrate to the activated copper-oxygen species. These 4-nitrocatechol results elegantly unify the catalytic behavior across all substrates studied. For catechol oxidation, nuclearity primarily governs pre-equilibrium binding affinity, while for OAP oxidative coupling; dicopper cooperativity becomes decisive during the multi-electron turnover phase. The NC control experiment confirms that the observed oxidase activities arise from the synergistic combination of strong substrate binding with dicopper-mediated redox cooperativity, rather than coordination alone^81^.

**Table 9:** Kinetic parameters for the reversible coordination of 4-nitrocatechol (NC) to both complexes in methanol at 25 °C.

	k_1_[M^− 1^ s^− 1^]	k_− 1_ [s^− 1^]	K_a_ [M^− 1^]	K_d_ 10^− 3^ M
$\:[Cu{S}_{4}{]}_{2}$	53.9 ± 5.1	0.62 ± 0.01	86.9	11.5
$\:\left[CutBu{S}_{4}\right]$	52.0 ± 1.9	0.36 ± 0.10	144	6.9

### Catalytic performance and comparison with literature models

The catalytic profiles of $\:[Cu{S}_{4}{]}_{2}$ and $\:\left[Cu\right(tBu{S}_{4}\left)\right]$ reveal distinct nuclearity dependencies across the two oxidase mimics. The binuclear complex demonstrates substantially higher phenoxazinone synthase activity (TOF = 2772 $\:{h}^{-1}$) compared to its mononuclear counterpart (TOF = 684 $\:{h}^{-1}$), whereas both systems exhibit comparable catechol oxidase performance under saturating conditions (TOF ≈ 1700–1730 $\:{h}^{-1}$). This dichotomy highlights that dicopper cooperativity exerts a more pronounced influence on the complex six-electron OAP oxidative coupling than on the simpler two-electron catechol oxidation^11,82^. For phenoxazinone synthase mimicry, the superior performance of $\:[Cu{S}_{4}{]}_{2}$ aligns with the mechanistic demands of APX formation, which requires sequential oxidation, C-N bond formation, and dehydration involving reactive quinone-imine/radical intermediates^11,82,83^. The confined dicopper environment facilitates O_2_ activation while promoting productive substrate coupling, whereas the mononuclear complex struggles with these multi-step transformations despite favorable initial binding^11,82,83^. In contrast, catechol oxidation reveals that both nuclearity classes sustain efficient turnover once substrate saturation is achieved, with the binuclear advantage manifesting primarily through enhanced pre-equilibrium binding affinity rather than intrinsically faster oxidation chemistry^84^. Table 10 places these sulfur-ligated systems against established copper CO/PHS mimics, positioning $\:[Cu{S}_{4}{]}_{2}$ as a particularly effective dual-function catalyst while $\:\left[CutBu{S}_{4}\right]$ represents a rare mononuclear thiolate complex with competitive catechol oxidase activity. The comparison underscores that sulfur-rich coordination environments support robust oxidase mimicry across nuclearity classes, but optimal phenoxazinone synthase performance correlates strongly with binuclear copper architectures.

**Table 10:** Selected literature benchmarks for CO and PHS mimics under broadly comparable aerobic conditions in MeOH at 25 °C.

Complex	Nuclearity	Donor set/ligand class	COx TOF (h^−1^)	PHS TOF(h^−1^)	Ref.
$\:[Cu{S}_{4}{]}_{2}$	Dinuclear	Thiolate (S_4_^2−^)	1730	2772	This work
$\:\left[CutBu{S}_{4}\right]$	Mononuclear	Bulky thiolate (tBuS_4_^2−^)	1690	684	This work
[Cu_2_(µ-thiolate)]	Dinuclear	Thiolate donor	6900 h^− 1^	N/A	^69^
[Cu(thiolate-pyrazole)]	Mononuclear	Thiolate/N-donor	850	N/A	^85^
[Cu_2_(µ-S)(N₃O)]	Dinuclear	Thiolate-bridged mixed donor	2100	N/A	^85^
[Cu(S₃N)]	Mononuclear	Thiolate-rich mixed donor	2560	N/A	^68^
[Cu_2_(µ-phenoxo)(N_4_O_2_)]	Dinuclear	Phenoxo-bridged N/O donor	1118	1500	^86,87^
Dinuclear (Cu_2_) and tetranuclear (Cu_4_) systems	Dinuclear and tetranuclear	Mannich-base phenolate (N/O donor)	~ 40–80	N/A	^24^

Values are reported as TOF where available; N/A indicates that the corresponding activity was not reported.

### Proposed catalytic mechanisms

On the basis of the combined kinetic, spectroscopic, electrochemical, and computational investigations, a unified mechanistic framework is proposed for the catechol oxidase (CO) and phenoxazinone synthase (PHS) mimetic activities of the present copper complexes. In both systems, the catalytic cycle is initiated by rapid and reversible formation of a catalyst–substrate adduct, characterized by the equilibrium constant K_1_ (k_1_/k_-1_), in agreement with the stopped-flow kinetic measurements. This step corresponds to inner-sphere coordination of the deprotonated substrate to the Cu(II) center and represents the fundamental entry point into the catalytic cycle. Consistent with the kinetic results, the distinct catalytic profiles of [CuS_4_]_2_ and [CutBuS_4_] can be rationalized in terms of nuclearity-dependent mechanistic pathways. In general, dicopper systems promote cooperative substrate activation and more efficient O_2_ reduction, whereas mononuclear copper complexes operate via stepwise one-electron processes [68,69,72,82–84].

In catechol oxidase mimicry (3,5-DTBC → 3,5-DTBQ), the catalytic cycle proceeds via initial coordination of catecholate to the copper center. For the dicopper complex [CuS_4_]_2_, a bridging catecholate intermediate is likely formed, enabling cooperative oxidation at the binuclear core. Such systems commonly involve substrate binding accompanied by metal–ligand reorganization and formation of bridging catecholate species, followed by oxidation through peroxide- or superoxide-derived intermediates to afford the quinone product [68,69,72,82–84]. The lower K_M_ value observed for [CuS_4_]_2_ is therefore consistent with more efficient productive binding and stabilization of reactive oxygenated intermediates during turnover. In contrast, catechol oxidation by the mononuclear complex [CutBuS_4_] is best described by a stepwise pathway involving sequential one-electron transfer processes. In this mechanism, substrate oxidation generates a Cu(I) species together with a semiquinone radical semiquinone* intermediate. These steps involve Cu(II)/Cu(I) redox cycling, which constitutes the fundamental electron-transfer process governing both catalytic pathways. Subsequent reaction with molecular oxygen regenerates the Cu(II) center and produces the quinone product. Although this pathway lacks the concerted redox cooperativity of the dicopper system, it remains catalytically competent, particularly under substrate-saturating conditions.

In both systems, the catalytic cycle is closed by reaction of Cu(I) with molecular oxygen, leading to partial reduction of O_2_ and formation of H_2_O_2_. This step is most plausibly mediated through activated oxygen species such as superoxo or peroxo intermediates [11,88,89]. The experimental detection of H_2_O_2_ as a reaction by-product is consistent with an oxidase-type mechanism involving dioxygen reduction rather than direct oxygen insertion.

In PHS mimicry (o-aminophenol → phenoxazinone), a similar initial coordination step is followed by one-electron oxidation to generate an aminophenoxyl radical intermediate. This species undergoes further transformation to yield a quinone-imine (o-BQMI) intermediate, which represents the key precursor in the formation of the phenoxazinone chromophore [90,91]. The quinone-imine subsequently reacts with a second molecule of o-aminophenol in a non-catalytic coupling step, followed by cyclization and further oxidation to produce the final phenoxazinone product. In this context, the dicopper complex [CuS_4_]_2_ facilitates both oxidation and coupling steps through cooperative interactions within the bimetallic core, thereby enhancing the overall efficiency of the multi-electron conversion. By contrast, although [CutBuS_4_] can effectively bind o-aminophenol in the initial step, the absence of a second nearby copper center limits the efficiency of the subsequent oxidative-coupling sequence, which is intrinsically more demanding. Consequently, nuclearity has only a modest influence on catechol oxidation under substrate-saturated conditions but plays a decisive role in the more complex phenoxazinone formation pathway.

**Fig. 11:**
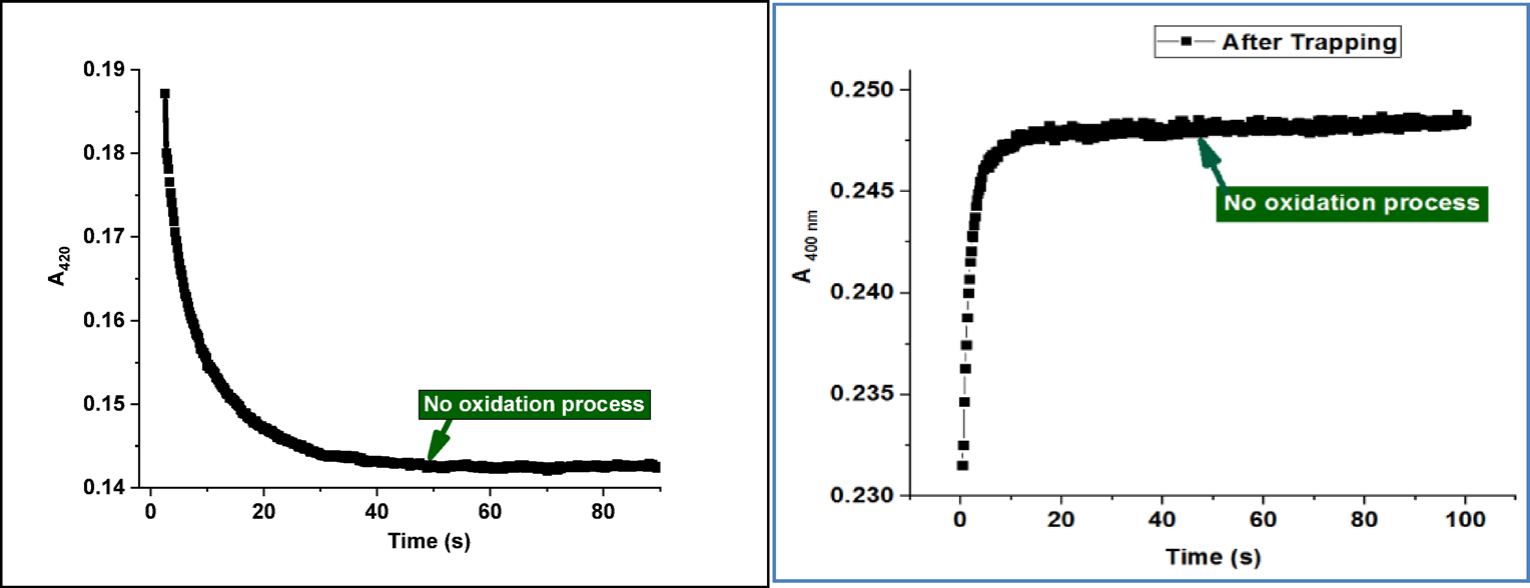
Radical-trapping experiments for the [CuS_4_]_2_ catalyst. Left: Time-dependent absorbance at 420 nm for the aerobic oxidation of OAP in methanol at 25 °C in the presence of isopropanol as a radical scavenger, showing strong attenuation of product formation. Right: Time-dependent absorbance at 400 nm for the aerobic oxidation of 3,5-DTBC under the same conditions, where the absence of a significant absorbance increase indicates suppression of the oxidation process after trapping.

To further support the proposed mechanisms, the involvement of radical intermediates was probed using radical-trapping experiments in the presence of isopropanol. The pronounced inhibition of OAP and 3,5-DTBC oxidation by isopropanol is illustrated in Fig. 11. Under otherwise identical conditions, the catalytic reactions exhibited a significant decrease in rate and attenuation of product formation, indicating suppression of the catalytic cycle. These observations provide experimental support for the participation of substrate-centered radical intermediates (aminophenoxyl/semiquinone) in the proposed mechanism. In addition, the quinone-imine structure is identified as a stable intermediate, corroborating its central mechanistic role in the PHS pathway.

**Scheme 3:**
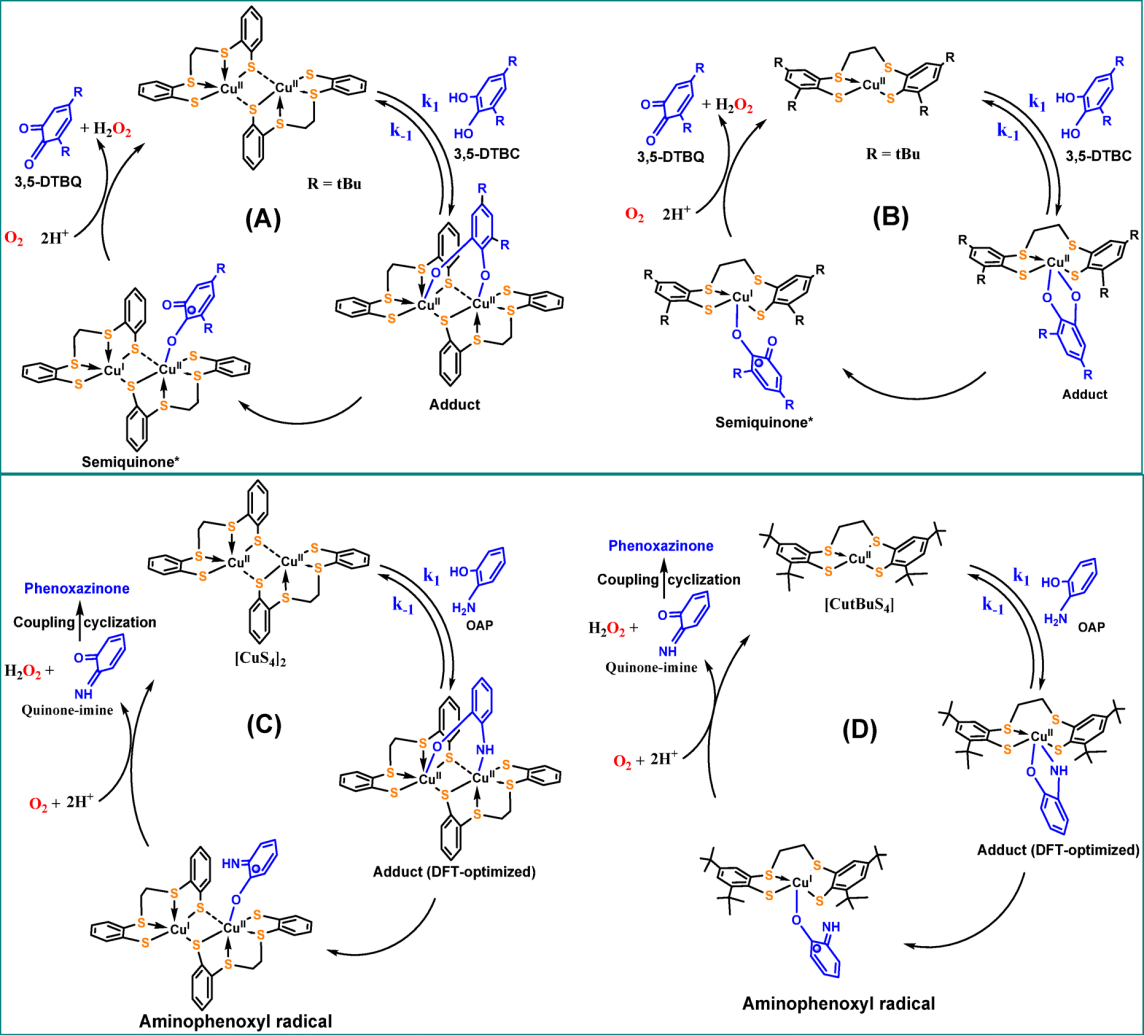
Proposed catalytic mechanisms for catechol oxidase (CO) (**A**,**B**) and phenoxazinone synthase (PHS) (**C**,**D**) mimic activities of mono- and dicopper complexes. Copper is reduced during substrate oxidation (Cu^II^ → Cu^I^) and reoxidized during O_2_ activation (Cu^I^ → Cu^II^), completing the catalytic cycle.

Overall, the catalytic cycles for both CO and PHS mimicry proceed via stepwise inner-sphere electron-transfer processes involving Cu(II)/Cu(I) redox cycling, substrate-centered radical intermediates, and activation of molecular oxygen. The observed differences in catalytic behavior arise primarily from the nuclearity of the copper center, which governs substrate activation, oxygen reduction, and, most critically, the multi-step oxidative-coupling sequence required for phenoxazinone formation [90,91] (Scheme 3).

### Nuclearity-dependent structure-reactivity relationships

The comprehensive dataset reveals how steric tuning within a common S_4_ ligand framework systematically modulates copper nuclearity, substrate binding, and catalytic selectivity. The binuclear $\:[Cu{S}_{4}{]}_{2}$ excels in phenoxazinone synthase mimicry through dicopper cooperativity during multi-electron oxidative coupling, while both complexes prove competent for catechol oxidation once substrate saturation overcomes binding differences. Electrochemical analysis confirms both systems access requisite Cu^II^/Cu^I^ redox states with 1:1 stoichiometry, yet the mononuclear complex’s higher formation constant (log $\:{K}_{f}$ = 13.18) highlights thermodynamic stabilization without corresponding catalytic enhancement. The 4-nitrocatechol control experiment provides the crucial distinction: strong substrate binding alone cannot drive turnover absent favorable electron-donor ability. Effective oxidase catalysis therefore demands synergistic interplay of productive coordination, substrate redox potential, and crucially for PHS activity, metal-metal cooperativity. These sulfur-ligated systems establish remote tert-butyl substitution as a powerful design lever that tunes nuclearity while preserving S-donor character, offering a modular platform for bioinspired oxidation catalysis.

## Conclusion

Remote tert-butyl substitution within a common tetrathioether–dithiolate ligand framework provides precise control over copper(II) nuclearity, cleanly switching from binuclear [CuS_4_]_2_ to mononuclear [CutBuS_4_]. This structural divergence systematically modulates electronic structure, thermodynamic stability, and catalytic selectivity, as established through combined structural, electrochemical, kinetic, and DFT analysis. Both complexes function as efficient catechol oxidase mimics with nearly identical maximum turnover frequencies (~ 1700 h^−1^) under substrate-saturated conditions, demonstrating that mononuclear thiolate units are fully competent for two-electron oxidase chemistry when binding limitations are overcome.

In contrast, a decisive nuclearity effect emerges in phenoxazinone synthase mimicry, where the binuclear system achieves a roughly four-fold higher turnover rate than the mononuclear analogue (2772 vs. 684 h^−1^), establishing dicopper cooperativity as essential for efficient multi-electron oxidative coupling. Detailed stopped-flow kinetics reveal complementary binding–turnover control: the binuclear complex excels in pre-equilibrium substrate coordination for catechol oxidation, while both nuclearities support comparable intrinsic oxidation rates. The 4-nitrocatechol control confirms that productive binding alone cannot drive turnover, and that favorable substrate redox potentials, together with, in the case of PHS activity, bimetallic cooperativity, are required.

Overall, these findings define a generalizable design principle: remote steric tuning within sulfur-donor platforms enables predictable control of nuclearity, substrate affinity, and pathway selectivity in type-3 copper enzyme mimics. The resulting binuclear–mononuclear pair thus represents a versatile, structurally well-defined platform for bioinspired oxidation catalysis, with promising opportunities for future extension to aqueous media, heterogeneous analogues, and other earth-abundant first-row metal systems.

## Supplementary Information

Below is the link to the electronic supplementary material.

Supplementary Material 1

## Data Availability

All data generated or analyzed during this study are included in this published article and its supplementary information files. Additional raw data supporting the findings of this study are available from the corresponding author on reasonable request.
