# Supplementary material for: Steric control of copper nuclearity in sulfur ligated oxidase mimics alters catechol and phenoxazinone oxidation
Source: Sci Rep. 2026 Jul 9;16:21432. doi: 10.1038/s41598-026-60865-4 (PMC13351046; doi:10.1038/s41598-026-60865-4)
Supplement: Supplementary file 1 — Supplementary Material 1 [file 41598_2026_60865_MOESM1_ESM.docx]

**
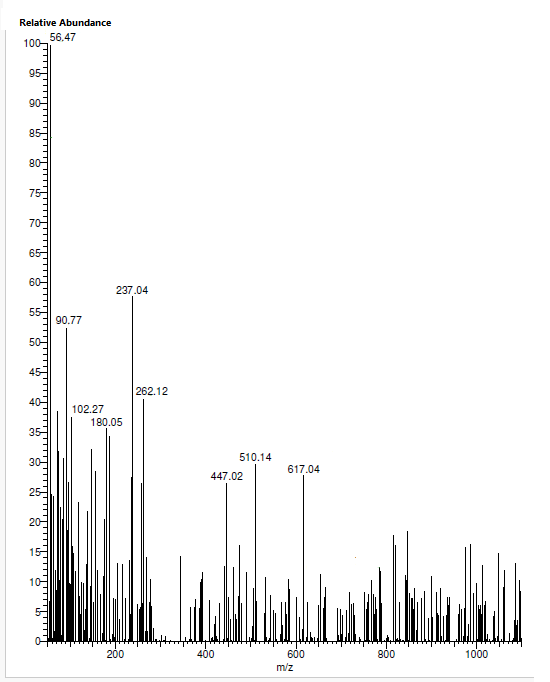
**

**Figure S1.** Electron ionization mass spectrum of the mononuclear complex [CutBuS₄].


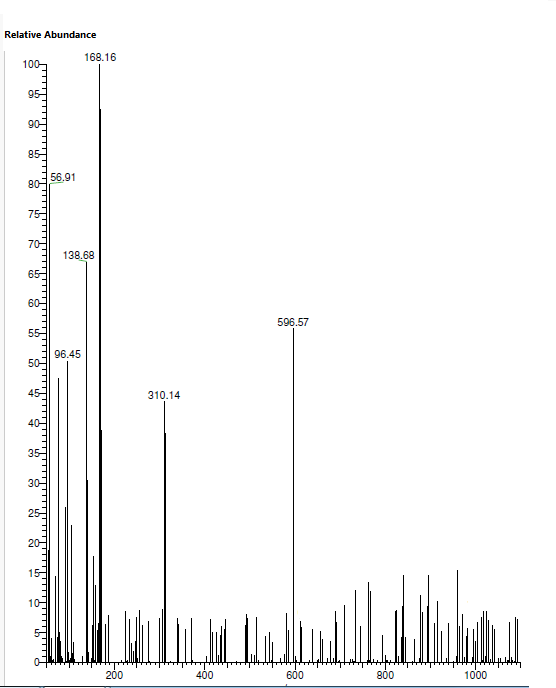


**Figure S2.** Electron ionization mass spectrum of the dinuclear complex [CuS₄]_2_.

Table S1. Cartesian coordinates of the optimized geometries for all ligands and complexes.

| **Structures** | **COORD** |
| --- | --- |
| 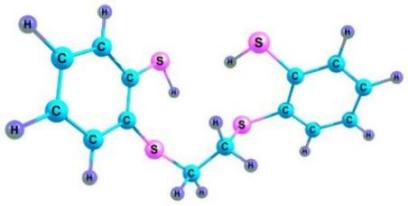  **S_4_-H_2_** | 16 -2.027239000 1.985530000 0.588703000  16 1.282719000 1.174060000 0.745455000  6 -4.315328000 -0.391907000 -1.769810000  6 -4.192712000 -1.646387000 -1.170665000  6 -3.407168000 -1.806991000 -0.036887000  6 -2.713798000 -0.721826000 0.522869000  6 -2.830811000 0.546357000 -0.085560000  6 -3.645555000 0.692636000 -1.216098000  6 2.773704000 0.406486000 0.153790000  6 3.929294000 1.192040000 0.062806000  6 5.134934000 0.653187000 -0.375191000  6 5.190109000 -0.694243000 -0.729924000  6 4.056749000 -1.492862000 -0.629285000  6 2.837192000 -0.964380000 -0.179915000  1 3.860501000 2.247094000 0.337663000  16 -1.749815000 -1.041499000 1.962528000  6 0.340498000 1.309924000 -0.817969000  6 -0.772034000 2.329983000 -0.689682000  1 -3.754948000 1.687727000 -1.655161000  1 -1.284014000 2.446827000 -1.657965000  1 -0.368523000 3.315360000 -0.406530000  1 1.041440000 1.636425000 -1.601918000  1 6.127803000 -1.134209000 -1.078373000  16 1.464415000 -2.062886000 -0.088972000  1 -0.052642000 0.320781000 -1.103442000  1 -4.718132000 -2.509306000 -1.586891000  1 -1.254829000 0.220357000 2.051423000  1 0.694049000 -1.268003000 0.700056000  1 -3.317683000 -2.791439000 0.429213000  1 4.111922000 -2.553736000 -0.886057000  1 -4.941261000 -0.256431000 -2.654385000  1 6.024581000 1.283158000 -0.441910000 |
| 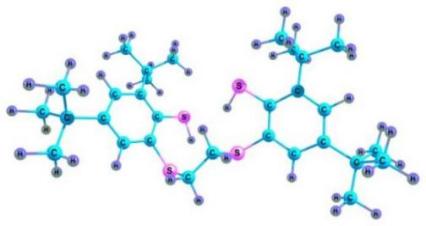  **tBuS_4_-H_2_** | 16 -1.677072000 -1.665850000 -1.798004000  16 1.544608000 -1.569913000 -1.407380000  6 -4.784320000 -1.158141000 0.730104000  6 -4.836688000 0.240910000 0.795026000  6 -3.977115000 1.109690000 0.117241000  6 -2.953769000 0.534039000 -0.699908000  6 -2.880907000 -0.876282000 -0.731601000  6 -3.784986000 -1.704667000 -0.066907000  6 2.855116000 -0.758110000 -0.525931000  6 3.970097000 -1.554285000 -0.243004000  6 5.064521000 -1.016693000 0.418271000  6 4.981655000 0.344321000 0.751963000  6 3.901795000 1.183058000 0.471320000  6 2.774973000 0.614846000 -0.207742000  1 3.953411000 -2.598999000 -0.556812000  16 -1.851360000 1.503142000 -1.686464000  6 0.474860000 -2.239004000 -0.068517000  6 -0.778529000 -2.825984000 -0.694603000  1 -3.702210000 -2.785491000 -0.191404000  1 -1.473372000 -3.177835000 0.081640000  1 -0.528784000 -3.685315000 -1.336034000  1 1.040779000 -3.017866000 0.466251000  1 5.835870000 0.780972000 1.267202000  16 1.343002000 1.555835000 -0.621491000  1 0.244287000 -1.422564000 0.633785000  1 -5.611751000 0.682395000 1.419179000  29 -0.064065000 0.123095000 -1.622967000  6 3.975306000 2.664170000 0.899467000  6 6.313986000 -1.825111000 0.776274000  6 -4.161438000 2.631461000 0.294685000  6 -5.798156000 -2.006198000 1.503193000  6 -5.650796000 -1.728532000 3.007615000  1 -4.641188000 -1.990565000 3.360313000  1 -5.822968000 -0.668524000 3.246816000  1 -6.379058000 -2.323163000 3.582477000  6 -7.221183000 -1.641724000 1.051256000  1 -7.964252000 -2.246300000 1.595859000  1 -7.452316000 -0.582724000 1.239396000  1 -7.352439000 -1.826563000 -0.026002000  6 -5.589573000 -3.503882000 1.267160000  1 -6.336501000 -4.078583000 1.835903000  1 -5.705784000 -3.772476000 0.205548000  1 -4.594954000 -3.838389000 1.601327000  6 -5.329003000 2.967902000 1.231908000  1 -5.181906000 2.566645000 2.246568000  1 -5.404443000 4.061983000 1.323007000  1 -6.295587000 2.605535000 0.848168000  6 -4.482128000 3.293085000 -1.056528000  1 -5.409688000 2.877597000 -1.481371000  1 -4.628205000 4.376167000 -0.915695000  1 -3.677576000 3.154651000 -1.790540000  6 -2.896955000 3.241680000 0.925008000  1 -2.000384000 3.047050000 0.322112000  1 -3.017476000 4.332717000 1.022031000  1 -2.733642000 2.828170000 1.932773000  6 3.886661000 3.578902000 -0.334594000  1 2.955392000 3.431888000 -0.897255000  1 3.932552000 4.634145000 -0.020883000  1 4.731585000 3.389496000 -1.015335000  6 2.854049000 2.980099000 1.904652000  1 2.973736000 2.374202000 2.816798000  1 2.899597000 4.042169000 2.194534000  1 1.856134000 2.783046000 1.490819000  6 5.299010000 2.999597000 1.598819000  1 6.170851000 2.840887000 0.945332000  1 5.291206000 4.063618000 1.879440000  1 5.444204000 2.419730000 2.523552000  6 6.509938000 -1.811383000 2.300502000  1 6.633794000 -0.789120000 2.688295000  1 5.645559000 -2.260823000 2.813478000  1 7.409154000 -2.384238000 2.579065000  6 6.201273000 -3.280934000 0.318248000  1 7.114766000 -3.830423000 0.592741000  1 5.351994000 -3.795995000 0.793781000  1 6.084963000 -3.360364000 -0.773817000  6 7.541442000 -1.198607000 0.095769000  1 7.428220000 -1.199661000 -0.999290000  1 7.700407000 -0.157528000 0.414413000  1 8.452225000 -1.766079000 0.346418000 |
| 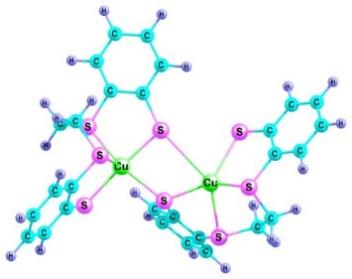  **[CuS_4_]_2_​** | 6 -4.649999000 0.522510000 -1.582558000  6 -4.446978000 1.482619000 -0.561275000  6 -5.356793000 2.561433000 -0.517285000  6 -6.406454000 2.673629000 -1.418056000  6 -6.587611000 1.717170000 -2.420388000  6 -5.698268000 0.651133000 -2.500431000  29 -1.885575000 -0.218532000 -0.194746000  16 -3.555086000 -0.873550000 -1.781409000  16 -3.184029000 1.408456000 0.646421000  16 -2.371822000 -3.486802000 0.240063000  6 -3.940463000 -2.570503000 0.455549000  6 -4.493277000 -2.190288000 -0.907794000  6 -1.758470000 -3.623268000 2.931004000  6 -1.454209000 -3.045998000 1.695375000  6 -0.371580000 -2.150767000 1.582203000  6 0.377718000 -1.849107000 2.725974000  6 0.054114000 -2.418459000 3.953472000  6 -1.015151000 -3.306233000 4.064163000  16 0.005355000 -1.464186000 -0.012255000  6 3.895280000 -0.834865000 -2.031229000  6 4.107464000 -1.741927000 -0.968276000  6 5.034921000 -2.782863000 -1.187119000  6 5.720821000 -2.904051000 -2.386948000  6 5.499052000 -2.000746000 -3.430182000  6 4.575872000 -0.979372000 -3.247063000  29 1.792838000 -0.018608000 0.398335000  16 2.686782000 0.466712000 -1.906822000  16 3.347200000 -1.661738000 0.606675000  16 3.188196000 1.625474000 1.350842000  6 4.352278000 1.769160000 -0.043113000  6 3.706691000 1.890507000 -1.408574000  6 2.819842000 4.363617000 1.570257000  6 2.241910000 3.135895000 1.236310000  6 0.881335000 3.037570000 0.901486000  6 0.111224000 4.213455000 0.892447000  6 0.695104000 5.435924000 1.201524000  6 2.049765000 5.521510000 1.537897000  16 0.132274000 1.483583000 0.539769000  1 -5.213648000 3.318273000 0.257400000  1 -7.088610000 3.524712000 -1.343930000  1 -7.402071000 1.809542000 -3.141993000  1 -5.803369000 -0.096311000 -3.291111000  1 -4.668297000 -3.217097000 0.972300000  1 -3.766121000 -1.673209000 1.070002000  1 -5.523457000 -1.816873000 -0.815183000  1 -4.511095000 -3.061734000 -1.581483000  1 -2.585864000 -4.334478000 2.989650000  1 1.214466000 -1.151058000 2.653381000  1 0.650027000 -2.163414000 4.833134000  1 -1.263699000 -3.757817000 5.027018000  1 5.204599000 -3.502763000 -0.383134000  1 6.431825000 -3.724394000 -2.515056000  1 6.025934000 -2.104050000 -4.380960000  1 4.360298000 -0.276477000 -4.056112000  1 4.996998000 2.639953000 0.156329000  1 4.969368000 0.859796000 0.036930000  1 4.486641000 2.038854000 -2.172049000  1 3.025245000 2.755924000 -1.450760000  1 3.873432000 4.406476000 1.859938000  1 -0.950640000 4.145071000 0.642726000  1 0.081325000 6.340114000 1.184488000  1 2.499341000 6.484758000 1.787614000 |
| 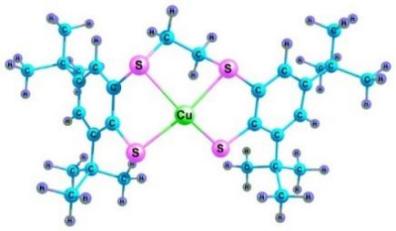  $\mathbf{[}\boldsymbol{CutBu}\boldsymbol{S}_{\mathbf{4}}\mathbf{]}$ | 16 -1.677072000 -1.665850000 -1.798004000  16 1.544608000 -1.569913000 -1.407380000  6 -4.784320000 -1.158141000 0.730104000  6 -4.836688000 0.240910000 0.795026000  6 -3.977115000 1.109690000 0.117241000  6 -2.953769000 0.534039000 -0.699908000  6 -2.880907000 -0.876282000 -0.731601000  6 -3.784986000 -1.704667000 -0.066907000  6 2.855116000 -0.758110000 -0.525931000  6 3.970097000 -1.554285000 -0.243004000  6 5.064521000 -1.016693000 0.418271000  6 4.981655000 0.344321000 0.751963000  6 3.901795000 1.183058000 0.471320000  6 2.774973000 0.614846000 -0.207742000  1 3.953411000 -2.598999000 -0.556812000  16 -1.851360000 1.503142000 -1.686464000  6 0.474860000 -2.239004000 -0.068517000  6 -0.778529000 -2.825984000 -0.694603000  1 -3.702210000 -2.785491000 -0.191404000  1 -1.473372000 -3.177835000 0.081640000  1 -0.528784000 -3.685315000 -1.336034000  1 1.040779000 -3.017866000 0.466251000  1 5.835870000 0.780972000 1.267202000  16 1.343002000 1.555835000 -0.621491000  1 0.244287000 -1.422564000 0.633785000  1 -5.611751000 0.682395000 1.419179000  29 -0.064065000 0.123095000 -1.622967000  6 3.975306000 2.664170000 0.899467000  6 6.313986000 -1.825111000 0.776274000  6 -4.161438000 2.631461000 0.294685000  6 -5.798156000 -2.006198000 1.503193000  6 -5.650796000 -1.728532000 3.007615000  1 -4.641188000 -1.990565000 3.360313000  1 -5.822968000 -0.668524000 3.246816000  1 -6.379058000 -2.323163000 3.582477000  6 -7.221183000 -1.641724000 1.051256000  1 -7.964252000 -2.246300000 1.595859000  1 -7.452316000 -0.582724000 1.239396000  1 -7.352439000 -1.826563000 -0.026002000  6 -5.589573000 -3.503882000 1.267160000  1 -6.336501000 -4.078583000 1.835903000  1 -5.705784000 -3.772476000 0.205548000  1 -4.594954000 -3.838389000 1.601327000  6 -5.329003000 2.967902000 1.231908000  1 -5.181906000 2.566645000 2.246568000  1 -5.404443000 4.061983000 1.323007000  1 -6.295587000 2.605535000 0.848168000  6 -4.482128000 3.293085000 -1.056528000  1 -5.409688000 2.877597000 -1.481371000  1 -4.628205000 4.376167000 -0.915695000  1 -3.677576000 3.154651000 -1.790540000  6 -2.896955000 3.241680000 0.925008000  1 -2.000384000 3.047050000 0.322112000  1 -3.017476000 4.332717000 1.022031000  1 -2.733642000 2.828170000 1.932773000  6 3.886661000 3.578902000 -0.334594000  1 2.955392000 3.431888000 -0.897255000  1 3.932552000 4.634145000 -0.020883000  1 4.731585000 3.389496000 -1.015335000  6 2.854049000 2.980099000 1.904652000  1 2.973736000 2.374202000 2.816798000  1 2.899597000 4.042169000 2.194534000  1 1.856134000 2.783046000 1.490819000  6 5.299010000 2.999597000 1.598819000  1 6.170851000 2.840887000 0.945332000  1 5.291206000 4.063618000 1.879440000  1 5.444204000 2.419730000 2.523552000  6 6.509938000 -1.811383000 2.300502000  1 6.633794000 -0.789120000 2.688295000  1 5.645559000 -2.260823000 2.813478000  1 7.409154000 -2.384238000 2.579065000  6 6.201273000 -3.280934000 0.318248000  1 7.114766000 -3.830423000 0.592741000  1 5.351994000 -3.795995000 0.793781000  1 6.084963000 -3.360364000 -0.773817000  6 7.541442000 -1.198607000 0.095769000  1 7.428220000 -1.199661000 -0.999290000  1 7.700407000 -0.157528000 0.414413000  1 8.452225000 -1.766079000 0.346418000 |


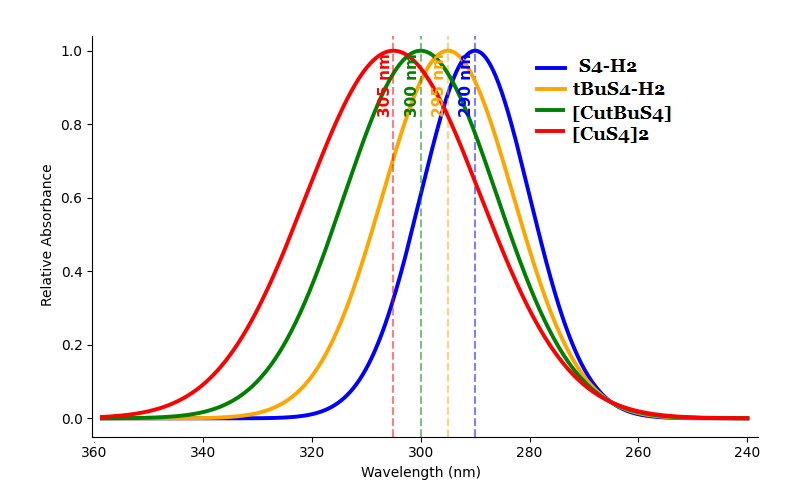


**Figure S3.** Simulated UV–Vis spectra (200–800 nm) (TD-PBE1PBE/def2-SVP); normalized for comparison.
